# Induction of Selective Blood-Tumor Barrier Permeability and Macromolecular Transport by a Biostable Kinin B1 Receptor Agonist in a Glioma Rat Model

**DOI:** 10.1371/journal.pone.0037485

**Published:** 2012-05-21

**Authors:** Jérôme Côté, Veronica Bovenzi, Martin Savard, Céléna Dubuc, Audrey Fortier, Witold Neugebauer, Luc Tremblay, Werner Müller-Esterl, Ana-Maria Tsanaclis, Martin Lepage, David Fortin, Fernand Gobeil

**Affiliations:** 1 Department of Pharmacology, University Hospital, Frankfurt, Germany; 2 Department of Nuclear Medicine and Radiobiology, University Hospital, Frankfurt, Germany; 3 Institute for Biochemistry II, University Hospital, Frankfurt, Germany; 4 Department of Pathology, Centre Hospitalier Universitaire de Sherbrooke, Université de Sherbrooke, Sherbrooke, Quebec, Canada; 5 Department of Surgery, Université de Sherbrooke, Sherbrooke, Quebec, Canada; 6 Institute of Pharmacology, Faculty of Medicine and Health Sciences, Université de Sherbrooke, Sherbrooke, Quebec, Canada; Julius-Maximilians-Universität Würzburg, Germany

## Abstract

Treatment of malignant glioma with chemotherapy is limited mostly because of delivery impediment related to the blood-brain tumor barrier (BTB). B1 receptors (B1R), inducible prototypical G-protein coupled receptors (GPCR) can regulate permeability of vessels including possibly that of brain tumors. Here, we determine the extent of BTB permeability induced by the natural and synthetic peptide B1R agonists, LysdesArg^9^BK (LDBK) and SarLys[dPhe^8^]desArg^9^BK (NG29), in syngeneic F98 glioma-implanted Fischer rats. Ten days after tumor inoculation, we detected the presence of B1R on tumor cells and associated vasculature. NG29 infusion increased brain distribution volume and uptake profiles of paramagnetic probes (Magnevist and Gadomer) at tumoral sites (*T*_1_-weighted imaging). These effects were blocked by B1R antagonist and non-selective cyclooxygenase inhibitors, but not by B2R antagonist and non-selective nitric oxide synthase inhibitors. Consistent with MRI data, systemic co-administration of NG29 improved brain tumor delivery of Carboplatin chemotherapy (ICP-Mass spectrometry). We also detected elevated B1R expression in clinical samples of high-grade glioma. Our results documented a novel GPCR-signaling mechanism for promoting transient BTB disruption, involving activation of B1R and ensuing production of COX metabolites. They also underlined the potential value of synthetic biostable B1R agonists as selective BTB modulators for local delivery of different sized-therapeutics at (peri)tumoral sites.

## Introduction

Major advances in medical and surgical treatments in the last few decades have increased the overall survival of patients with many types of cancers with the exception of those in the central nervous system (CNS) [1], [2]. In fact, the survival of patients with malignant gliomas more precisely glioblastoma (WHO grade IV), the most aggressive and prevalent primary brain tumors in adults (about 20–30%), has remained virtually unchanged over the last 40 years [3]. Furthermore, analytical epidemiologic studies indicate that the incidence of these tumors is steadily increasing in children and adults [4], [5]. The cause of this increase in incidence currently remains unknown. High grade malignant gliomas (WHO grades III and IV) are characterized by high levels of proliferative, migratory and invasion activities as well as high resistance to treatment. These hallmarks typify the aggressive tumor phenotype and account for the very poor prognosis and as yet, the incurable nature of the disease. Treatments of patients with malignant gliomas thus remain palliative and generally include surgery, radiotherapy, and chemotherapy in various combinations. Chemotherapy is assuming an increasingly important role in the treatment of malignant gliomas [6], [7], [8]. Chemotherapeutic agents are commonly administered orally or intravenously [9]. However, these routes of administration do not provide high concentrations of drugs in the brain parenchyma especially because of the blood-brain barrier (BBB) that isolates the brain from the rest of the body [10]. It has been estimated that only about 2% of drugs pass through this barrier [11], [12]. The impermeability of the BBB is due to tight junction proteins connecting adjacent endothelial cells, which inhibit any significant paracellular transport. The highly regulated transport systems of the endothelial cell membranes also restrain drug transcytosis across the BBB (see review [13]). The BBB in malignant brain tumors (also referred to as blood-tumor barrier (BTB)) is abnormal and variably disrupted within the main body of the tumor and nearby tissue. Various parts of tumors, especially large areas of diffuse infiltrative tumors, with a mainly intact BTB may thus be shielded from chemotherapy [14]. There is thus a critical need to develop successful methods to safely open the BTB in order to improve chemotherapeutic treatment for malignant glioma.

Kinins are a group of autacoid peptides formed by numerous tissues and in the blood at the vascular endothelial layer. They are natural modulators of the tone and permeability of vessels including the cerebral microcirculation [15], [16]. Bradykinin (BK) and kallidin (LysBK), and their respective bioactive natural metabolites (desArg^9^BK and LysdesArg^9^BK), which lack the C-terminal arginyl residue, are the main sources of kinin activity. The biological effects of BK- and desArg^9^-related peptides are mediated through the activation of specific GPCRs called B2R and B1R, respectively [17]. Like most GPCRs, B2R exhibit constitutive expression with measurable levels of these receptors under normal conditions. B2R is thought to be responsible for most of kinin activities under physiological conditions, including the regulation of the cardiovascular and kidney functions [17]. On the other hand, B1R is inducible and is expressed in major inflammatory pathologies such as cardiovascular diseases and cancer [17], [18], making it an attractive pharmaceutical target with anticipated reduced collateral effects. B1R is induced or overexpressed during tissue injury or ischemia, or following exposure to bacterial endotoxins or inflammatory cytokines such as interleukin-1β (IL-1β) and tumor necrosis factor α (TNF-α) [18]. Gliomas, like many other solid tumors, are surrounded by a zone of inflammation that is needed for sustained tumor growth and angiogenesis [19], [20]. This process is partly dependent on glioma tumor derived cytokines IL-1β and TNF-α [19], [21], which could plausibly trigger expression and activity of B1R impacting on the brain tumor microcirculatory system. Preliminary experiments revealed that tumor F98 gliomas implanted in the brains of rats have high levels of IL-1β immunoreactivity, providing support to this hypothesis (Figure S1). Furthermore, in vivo studies on the effects of exogenous pharmacological agonists and antagonists in a number of animal disease models have revealed that B1R may play a role in inducing systemic vascular permeability in peripheral organs and have been generally supported by semi-quantitative Evans blue analyses [22], [23], [24], [25], [26]. In central nervous tissue, B1R also appears to modulate BBB permeability, including that of brain tumors [27], [28], [29]. This is consistent with preliminary clinical observations from our group and others showing that endothelial and glioma cells in human malignant glioma specimens exhibit B1R immunoreactivity [30], [31]. The exact role of B1R in the glioma biology remains unclear. Although the evidence so far seems to be pointing to a vasomodulator role of B1R in the brain tumor vasculature, no clear and convincing evidence has yet been established.

Based on this rationale, we surmised that selective B1R agonists, when infused systemically, would induce selective BTB disruption thereby maximizing macromolecular delivery and efficacy of the chemotherapeutic agents used to treat malignant glioma. In addition, agonist treatments would not result in systemic complications because of the restricted expression of B1R target in the glioma environnment. We used syngeneic F98 glioma bearing rats as a clinically relevant animal model of malignant brain cancer [32], [33]. Our objectives were 1) to detect and locate B1R expression in rat and human brain tumor (for clinical validation purposes) by integrating molecular and cellular biology approaches, and 2) to correlate B1R expression to in vivo functional permeability data of peptide agonists that selectively target B1R. Previous studies, including ours, have shown that non-invasive dynamic contrast-enhanced magnetic resonance imaging (DCE-MRI) is a reliable method to determine the spatio-temporal BTB opening in vivo [34], [35], [36], [37], [38], [39]. We thus used this technique with gadolinium-based contrast agents Magnevist (Gd-DTPA; 0.5 kDa) and Gadomer (17 kDa) as brain intravascular tracers, together with conventional immunohistochemistry (IHC) and Evans blue staining of albumin (∼65 kDa), to assess the extent and duration of BTB permeability. We also explored whether a B1R agonist can enhance bioavailability of the chemotherapeutic agent Carboplatin (and Magnevist used as reference) in brain tumor tissues using inductively coupled plasma-mass spectroscopy (ICP-MS). The natural B1R agonist LysdesArg^9^BK (LDBK) and its degradation resistant, long-acting analogue SarLys[dPhe^8^]desArg^9^BK, NG29 [40], capable of improved stimulation of BTB opening, were used for comparison purpose.

Here, we report that NG29, and probably other synthetic biostable B1R agonists sharing the proper pharmacokinetic features [40], can be used as selective BTB modifiers to improve transvascular delivery of various-sized water soluble molecules to CNS tumors expressing substantial level of B1R. These results may constitute a significant contribution to the development of effective systemic chemotherapy modalities for the treatment of inoperable or of recurrent malignant brain tumors.

## Results

### Kinin B1R are Overexpressed in Rat Brain F98 Glioma Tissue, in Human Glioma Cell Lines and in Human Glioma Specimens

**Figure 1:**
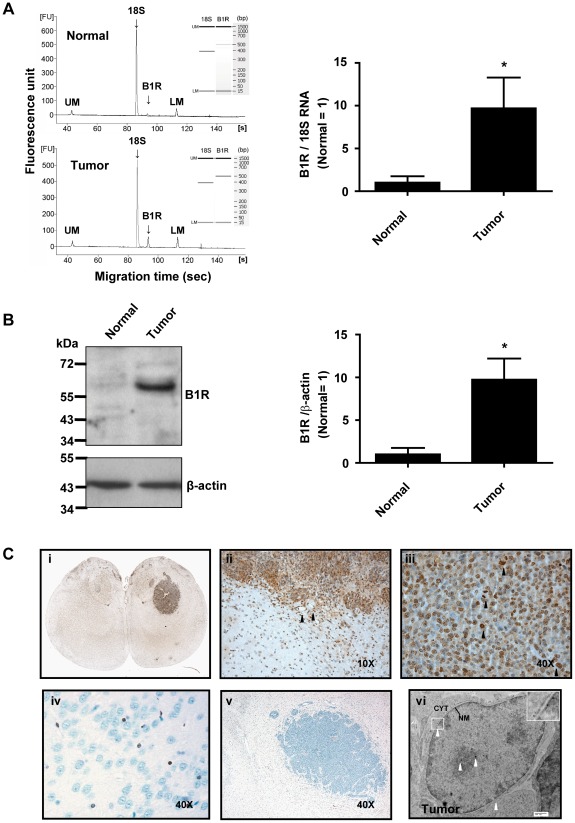
B1R expression in normal cerebral cortical and tumoral tissues of F98 glioma-bearing rats. (**A**) Left: Representative electropherograms and gel-like images (insets) of RT-PCR products amplified from one tumor and autologous controlateral tissue (normal); LM and UM correspond to lower and upper internal markers, respectively. Right: Histographic representation of B1R expression from multiple normal and tumoral tissues. B1R mRNA level was normalized to the corresponding 18S level for each biopsy. n = 5 rats. *p<0.05 vs normal. (**B**) Western blot of rat brain soluble protein extracts probed with the anti-B1R antiserum AS434. Left: Rat brain tumor shows a robust single immunoreactive band around 60 kD. In the absence of antiserum, no band was seen in the rat brain samples (not shown). Right: Histographic representation of B1R expression from multiple normal and tumoral tissues. B1R protein level was normalized to the corresponding β-actin level for each biopsy. n = 4 rats. *p<0.05 vs normal. (**C**) The antiserum used for Western blot was also used to characterize the location of the proteins in rat brain cortical samples by IHC, along with HRP (panels i–v), and TEM immunogold labeling (panel vi). Photomicrographs illustrating positive B1R immunoreactivity in tumor (**C**, i–iii) and microvascular endothelial cells (ii) adjacent to the tumor (black arrowheads). Note the chromosomal staining on all tumor cells under mitosis (black arrowheads, iii). Negative control with preimmune serum showed no staining (v). Weak positive B1R staining of glial cells from both implanted (ii) and contralateral hemispheres (iv) is also shown. Magnification as indicated. EM photomicrograph (vi) showing subcellular localization of B1R in a glioma cancer cell (white arrowheads). Insert: digital enlargement of the delineated area (white rectangle) showing B1R immunoreactivity at both the inner and outer leaflets of the nuclear envelope. Scale bar = 500 nm. Photomicrographs of HRP labeling were equalized in terms of contrast, brightness and gamma using ImagePro Plus 5.1.

We analysed the expression profile of B1R in rat brain normal and tumor tissues by RT-PCR and WB (Figure 1A, B). B1R mRNA and protein expression levels were significantly higher (p<0.05) in brain tumor tissues than in normal brain tissues. In fact, most normal tissues had very low to negligible levels of B1R. To confirm these results and provide an idea of the subcellular localization of B1R in glioma tissues, we performed standard peroxidase-based IHC. Glioma tissues and perivascular tumor microsatellites distant from the primary tumor mass were nearly all B1R immunoreactive (Figure 1C, ii). Notably, the positive staining with the anti-B1R antiserum AS434 was mainly intracellular (with few cell plasma membrane associated labeling) and was confined to perinuclear zones and inside the nucleus. Closer examination by high magnification transmission electron microscopy (TEM) confirmed the predominant localization of B1R to the endoplasmic reticulum (ER)/nuclear envelope and nucleoplasm of F98 glioma cells (Figure 1C, vi). Similar TEM results were obtained with the anti-B1R antibody RC72 (data not shown). We also detected moderate positive staining in intra- and peritumoral microvessels (Figure 1) and, sporadically, in ipsy- and contralateral normal glial cells. Staining was absent or barely detectable in neurons (Figure 1).

**Figure 2:**
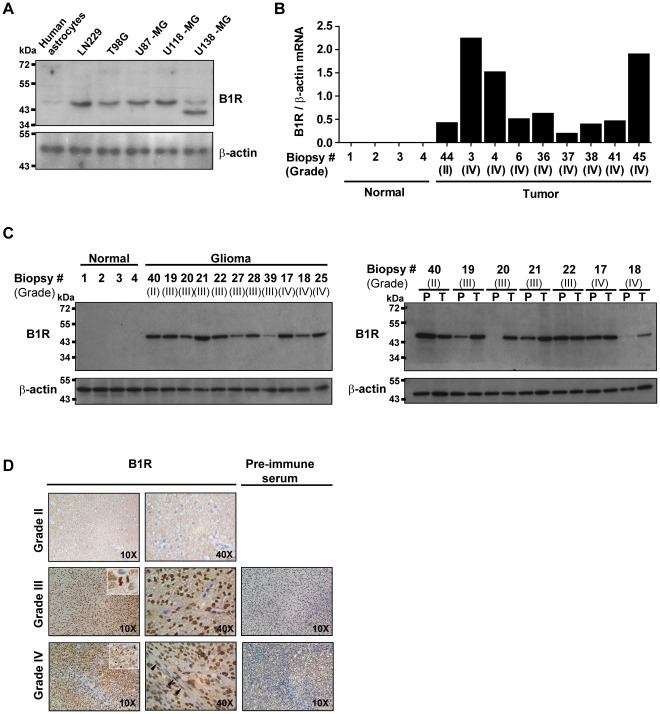
Overexpression of B1R in human glioma tissues. (**A**) Detection of B1R in various human glioma cell lines and nontransformed counterparts, normal human astrocytes, by WB analysis using the anti-B1R antibody RC72. β-actin serves as a loading control in the lower panel. The doublet band in U138-MG cells may indicate degradation of B1R. (**B**) Comparative quantification of B1R mRNA levels among normal and glioma brain tissue samples was normalized against that of the corresponding β-actin. (**C**) Expression of B1R in normal versus tumoral tissue specimens (left panel) or in paired primary glioma (T) and peritumoral tissue biopsies (P) (right panel), with each pair obtained from a same patient. Western blot analysis was performed using the anti-B1R antiserum AS434. (**D**) Representative images from IHC assay of paraffin-embedded specimens of primary glioma tissue biopsies (WHO grades II–IV) exposed to pre-immune serum or AS434 antiserum. Optical magnification is indicated in the bottom-right corner of each image.

In an initial effort to translate the results from the animal model to humans, we also examined the expression of B1R in well-established human glioblastoma cell lines, clinical glioma specimens of varying grades, and post-mortem normal control human brain biopsies (Figure 2A–D) using the same techniques used to quantify B1R expression profiles in rats. WB analysis consistently revealed significantly higher B1R protein expression in glioma cells relative to their nontransformed counterparts, normal human astrocytes (Figure 2A). The predominant B1R immunoreactive species (∼45 kDa) appearing in human glioma cells had a lower molecular-mass than that identified in rat glioma tissues (∼65 kDa) (Figure 1B), possibly due to differences in post-translational processing of B1R (ex. glycosylation). The striking in vitro upregulation was also revealed by RT-PCR (Figure 2B) and WB analyses (Figure 2C) of B1R in ex vivo human glioma biopsies. B1R transcripts were variably upregulated in human grade II-IV astrocytic tumors (9 cases) compared to normal brain cortical specimens (Figure 2B). WB analyses of a different panel of tumor tissues (11 cases) confirmed that B1R was expressed in tumors but not in normal brain tissue (Figure 2C, left panel). Moreover, WB analyses of protein extracts from seven pairs of matched human glioma tissues and peritumoral brain tissues (Figure 2C, right panel) showed that B1R was expressed in all glioma tissues analyzed while expression levels were variable in the inflamed peritumoral areas (4/7 cases). The WB results obtained with AS434 were validated using two other anti-B1R antibodies (LS799 and RC72), which generated very similar staining patterns (Figure S2).

Four cases of malignant human astrocytoma specimens that included one WHO grade 3 and three WHO grade 4 glioblastoma multiforme, tested positive for B1R expression using the IHC method (Figure 2D). Like to rat F98 glioma IHC staining, B1R-positive cells were mainly observed in tumor tissues and were mostly restricted to the perinuclear envelopes and within nuclei (Figure 2D). Moreover, B1R immunoreactivity was always associated with mitotic chromosomes in the anaplastic high-grade gliomas (Figure 2D, inset), likely due to the localization of B1R in fragmented endoplasmic reticulum (ER)/nuclear membranes wrapped around chromatins during mitosis [41]. Microvessels in malignant tumor also tested positive, albeit to lesser extent (Figure 2D, black arrowheads), but showed less to no nuclear staining. On the other hand, one case of benign diffuse low grade astrocytoma (WHO grade 2) stained negative for B1R (Figure 2D). These results provide support for the hypothesis that B1R might be a novel, valid pharmacological target for detecting malignant astrocytoma, manipulating newly forming vasculature integrity, and regulating tumor permeability using potent B1R agonists in preclinical and clinical settings. However, given the small sample size for human subjects and possible risk of unrepresentative astrocytoma biopsy sampling related to intratumoral genetic heterogeneity [42], further work is required to determine the appropriateness of these results.

### The Peptide Agonist NG29 Induces Effective Transvascular Delivery Across the BTB of Malignant Glioma and the Accumulation of Two Different-sized Contrast Agents via B1R and a COX-dependent Pathway

**Figure 3:**
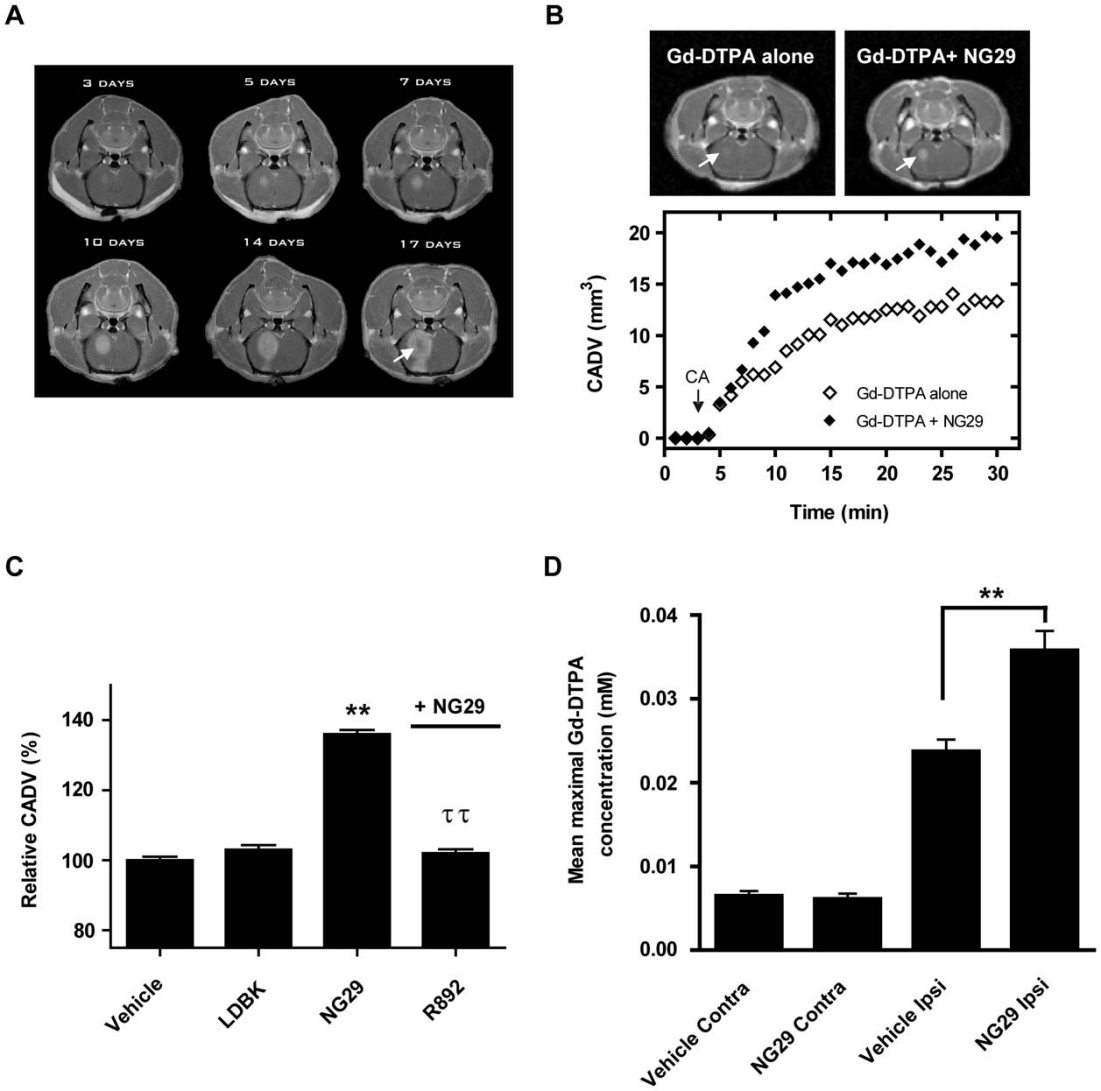
NG29 enhances transvascular delivery, distribution and accumulation of Magnevist within brain tumor tissues of F98-bearing rats. (A) MRI contrast-enhanced detection of glial brain tumors in rats at day 3, 5, 7, 10 14 and 17 post-inoculation. Note the rapidly growing tumor over a 2-week time and the appearance of a necrotic center on the 17 day-image (white arrow) outgrowing its blood supply. Assessment of BTB disruption by MRI monitoring was conducted on the same animal on day 10 post-inoculation, corresponding to mid stage development of the tumor. (B) Representative axial Magnevist-enhanced *T*_1_-weighted MR images depicting the brain of an F98-implanted rat before and after NG29 treatment (10 nmol/kg/min for 5 min i.c.) (left panel). Note the increase in the signal intensity at the tumor (white arrows). Temporal CADV calculated from the corresponding sets of images (1 image/51 s for 50 min) (bottom panel). (C) Relative CADV in percent determined following the infusion of the vehicle (saline), LDBK, NG29 (10 nmol/kg/min for 5 min) or NG29 (10 nmol/kg/min, 5 min) + R892 (20 nmol/kg/min, 5 min). Each bar represents the mean ± S.E.M. for 4 to 6 animals. **p<0.01 compared to vehicle-treated animals; ^††^p<0.01 compared to NG29-treated animals. (D) Histographic representation of average maximal Gd-DTPA concentrations in the ipsilateral (tumor-implanted) and the contralateral hemispheres following saline vehicle or NG29 treatment (10 nmol/kg/min)). Note the superior levels of Gd-DTPA (reflecting greater basal permeability) in the ipsi- versus contralateral tissues of vehicle-treated animals (not illustrated, ***p<0.001). **p<0.01 compared to vehicle-treated ipsilateral groups. Value represents the mean ± S.E.M. obtained with 3 animals.

**Figure 4:**
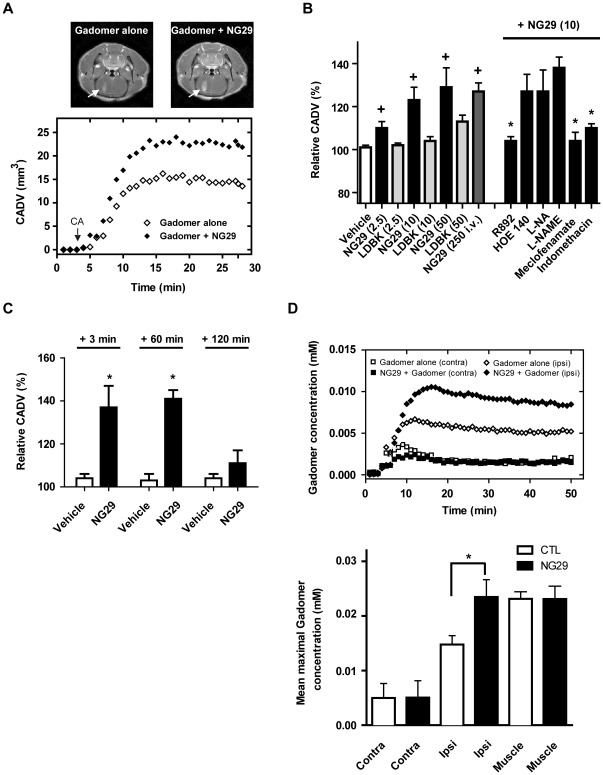
NG29 increases transvascular delivery, distribution and accumulation of Gadomer within brain tumor tissues of F98-bearing rats. (A) Representative axial Gadomer-enhanced *T*_1_-weighted MR images depicting the brain of an F98-implanted rat before and after intracarotid NG29 treatment (10 nmol/kg/min for 5 min i.c.) (upper panels). Note the increase of the signal intensity at the tumor (white arrows). CADV in function of time calculated from the corresponding set of images (1 image/51 s for 50 min) (bottom panel). (B) Dose-, B1R-, PGs-dependence of NG29-induced BTB permeability. Numbers in parenthesis represent infusion rates in nmol/kg/min, for 5 min. The B2R antagonist HOE140 and the B1R antagonist R892 (both at 20 nmol/kg/min, for 5 min, i.c.) were infused simultaneously with NG29 while the non-selective nitric oxide synthase (NOS) inhibitors L-NA (5 mg/kg, i.v.) and L-NAME (20 mg/kg, i.v.), and the non-selective cyclooxygenase (COX) inhibitors Meclofenamate (5 mg/kg, i.v.) and Indomethacin (2.5 mg/kg, i.v.) were administered 30 min before the infusion of NG29. Note the effectiveness of NG29 administered by the i.v. (intrajugular) route. ^+^p<0.05 compared to vehicle-treated animals; *p<0.05 compared to NG29 (10 nmol/kg/min)-treated animals. (C) Duration of the increase in BTB permeability caused by NG29 as detemined by relative CADV values. Gadomer was injected 3 min, 60 or 120 min following the start of the infusion (10 nmol/kg/min) of NG29 over 5 min. Each bar represents the mean ± S.E.M. for 3 to 7 animals. *p<0.05 compared to respective vehicle-treated animals. (D) Representative time course of Gadomer uptake in the ipsilateral (tumor-implanted) hemisphere and the contralateral hemisphere, before and after treatment with NG29 (10 nmol/kg/min for 5 min) (left panel). Histographic representation of average maximal Gadomer concentrations in the ipsilateral (tumor-implanted) and the contralateral hemispheres, and the facial muscle following NG29 treatment (right panel). As observed for Gd-DTPA, levels of Gadomer were higher in the ipsi- (tumors) than in contralateral (normal) tissues in CTL animals (not illustrated, ***p<0.001). *p<0.05 compared to untreated (Gadomer alone) ipsilateral groups. Value represents the mean ± S.E.M. obtained from 3 animals.

Having shown that B1R are overexpressed in rat brain F98 tumors and its associated blood vascular network, we then used real-time MRI with two different sized-CA (Magnevist (Gd-DTPA; 0.5 kDa) and Gadomer (17 kDa)) (Figures 3 and 4) to determine localization and volume of the tumors and to provide non-invasive assessment of cerebral microvascular responses and BTB disruption to the natural B1R agonist LDBK and its analogue NG29. Magnevist and Gadomer are believed to be non-toxic, non-actively transported, and are regarded as usefull intravascular hydrophilic tracers for monitoring paracellular permeability (via inter-endothelial clefts) and extent of BBB disruption in vivo [34], [43]. The Magnevist-enhanced images revealed the presence of F98 gliomas as early as 3 days after their inoculation in rats, with changes in *T*_1_ signal intensities that became much stronger on follow-up through to day 17 (Figure 3A). This suggested a leaky and penetrable BTB, even during an early stage of tumor development. The tumor *T*_1_-weighted intensity was isointense with normal brain before injection of CA (data not shown). Compared with F98-implanted rats, we observed no Magnevist enhancement at the tumor sites on day 10 post-inoculation in rats that underwent sham surgery and that were inoculated with F98-free DMEM (data not shown). The signal enhancement of Magnevist (Figure 3B, upper panels) and of Gadomer (Figure 4B, upper panels) was more pronounced in the central core and the peripheral rim of the tumor relative to baseline following intracarotid (i.c.) infusion of NG29 (10 nmol/kg/min for 5 min), which translated to a larger maximum CA distribution volume (CADV) (Figures 3B and 4A, bottom panels). In fact, a dose-response relationship and a significantly higher average maximal CADV were observed with the i.c. NG29-treated group of animals (Magnevist: 36±3%; Gadomer: 23±6%) (Figures 3C and 4B). I.V. administration of NG29 also increased the total interstitial distribution volume of Gadomer in the tumor microenvironment but required obviously a higher dose to produce an effect similar to that seen with i.c. administration, which creates a rapid “first pass” effect in the brain tumor (Figure 4B). The increase of mean maximal CADV was not observed when the vehicle or an equimolar amount of the natural agonist LDBK was infused (Figures 3C and 4B). The relatively low-level disruption of the BTB induced by LDBK may be explained by the activity of kinin-destroying enzymes namely angiotensin-converting enzyme (ACE,), neutral endopeptidase 24.11 (NEP 24.11), endopeptidases 24.15 and 24.16 (EP24.15 and EP24.16), which are widely distributed in microvessels and in many tissues including the brain [44].

To investigate which kinin receptor-subtype mediates NG29 responses, the animals were treated with a combination of NG29 and the biostable B1R antagonist R892 [45] or the B2R antagonist HOE-140 [46] (in dose exceeding twice the agonist dose) (Figures 3C and 4B). As expected from previous *in vitro* selectivity studies [40], only R892 significantly blunted the NG29-induced increase in mean maximal CADV (Figures 3C and 4B). To elucidate the mechanism by which NG29 modulates the disruption of the BTB, we tested two series of inhibitors that block either the NOS or COX pathway (Figure 4B). The NOS inhibitors L-NA and L-NAME had no effect on NG29-induced disruption of BTB or changes in CADV. Conversely, pretreatment with meclofenamate or indomethacin, two structurally unrelated COX inhibitors, blocked the effects of NG29 (Figure 4B). This strongly indicated that COX byproducts (most probably PGI_2_ and/or PGE_2_) [15], [47], [48] play a role in regulating permeability of BTB. We investigated the reversibility and duration of action of i.c. NG29 by administering Gadomer at different times (3, 60 and 120 min) after the initiation of agonist infusion and determined mean maximal CADV as described above (Figure 4C). The time-course results showed that BTB modulatory responses to NG29 promptly peaked at 3 min, remained stable for at least 1 h then fade away completely after approximatively 2 h, indicating that the integrity of the BTB had been restored (Figure 4C).

Lastly, increases in CADVs elicited by i.c. NG29 (Figures 3C and 4B) were associated with increases in the apparent amount of CA crossing into the brain tumor interstitial space (Figures 3D and 4D). Differences in representative Gadomer concentration–time plots generated using i.c. NG29 between ipsilateral (tumor-implanted) and contralateral hemispheres can be appreciated in Figure 4D (upper panel). The average maximal Gadomer concentration following i.c. NG29 treatment into the ipsilateral compartment significantly exceeded that of control untreated group (Figure 4D, bottom panel). Similar results were obtained with Magnevist (Figure 3D). There was no tendency of increased Gadomer concentration over time in the contralateral hemisphere and the jaw muscle upon NG29 treatment, demonstrating the tumor-site specific activity of NG29 (Figure 4D).

### Comparison between the Intracerebral Delivery of Magnevist and Carboplatin Following Intraarterial or Intravenous NG29 Administration

**Figure 5:**
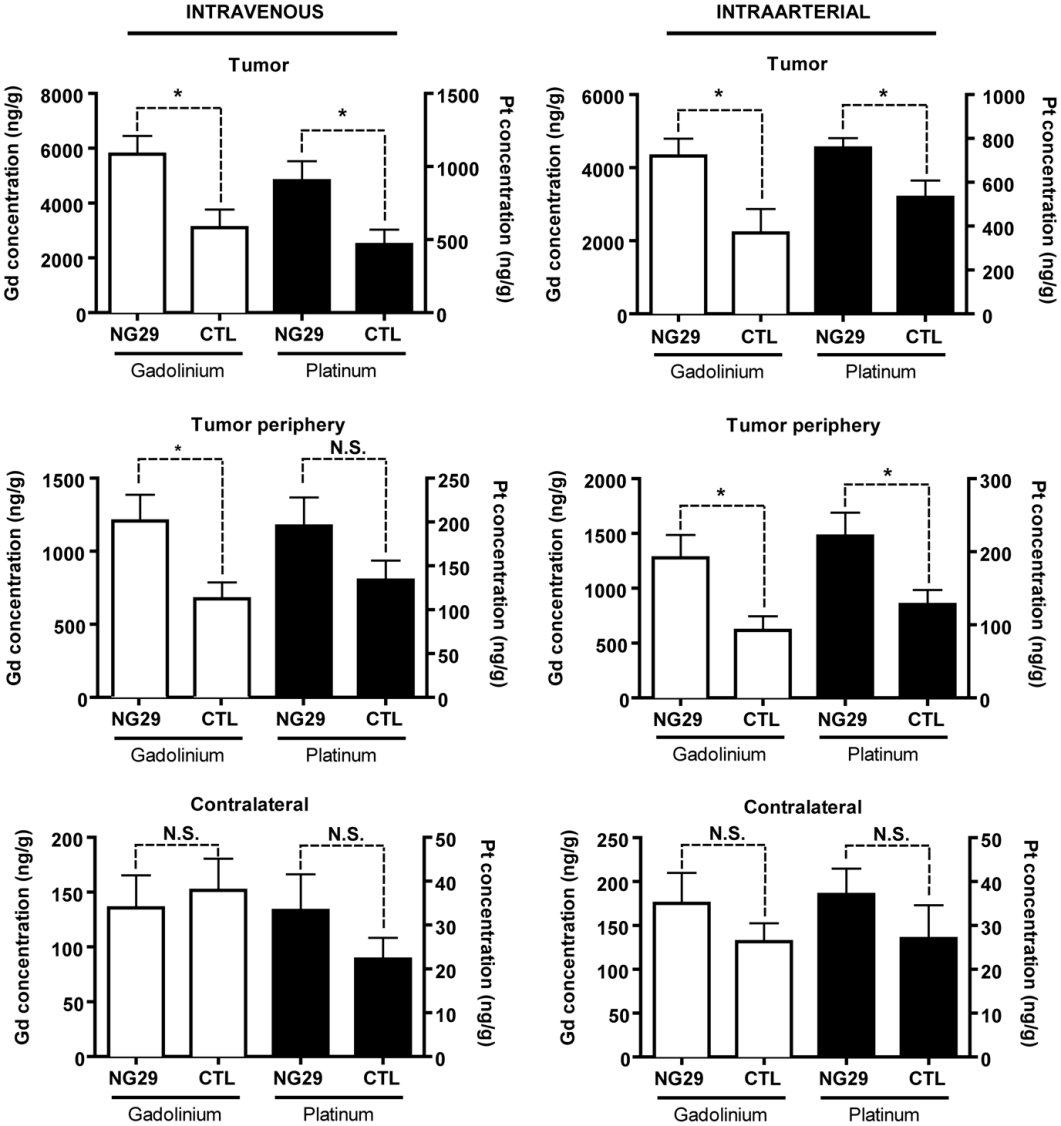
NG29 induces changes in local concentration of gadolinium and platinum in tumoral cerebral tissues. Direct measures of drug concentration (ng/g of tissue) by ICP-MS in three different tissue extracts (tumor, tumor periphery and contralateral) from the brain of F98 glioma-bearing Fischer rats were made following intraarterial (carotid artery) or intravenous (femoral vein) injections of Gd-DTPA (Gd) (143 mM i.v.) and carboplatin (Pt) (20 mg/kg i.a. or i.v.) with the B1R agonist NG29 (250 nmol/kg i.a.; 5 µmol/kg i.v.) or saline (CTL). Note the difference in ordinate scaling between drug concentrations in contralateral and tumoral tissues. Data are mean ± S.E.M. of 7–10 rats in each group. *p<0.05 versus respective control; N.S.: non significant.

To corroborate the contrast-enhanced MRI findings and to gain better insights into the potential of B1R agonists for delivering chemotherapeutic agents into CNS tumors via different routes of administration, we used the validated, highly sensitive, elemental ICP-MS method. We determined the Carboplatin platinum and Magnevist gadolinium content of tissue extracts from tumor, adjacent brain tissue, and cortical contralateral tissues (Figure 5). Even though it is known that F98 glioma cells are highly resistant to DNA-alkylating agents such as Carboplatin [49], we chose to study this chemotherapeutic agent because it is used to treat glioma patients, albeit with limited evidence of efficacy [50]. Its molecular size (371 Da) and hydrophilicity are comparable to Magnevist (500 Da). As such, we anticipated that its intracerebral bioavailability would be similar to that of Magnevist. Our results proved the hypothesis and showed that the systemic co-administration of NG29 with Magnevist and Carboplatin significantly enhanced their brain delivery and actual bona fide concentrations, to a similar degree, in tumor and peritumoral sites (∼2-fold increase in both cases) (Figure 5). Notably, the NG29/drug combination can be given i.v. or i.c. with similar effectiveness, taking into account dosage adjustement for difference on the administration site of the mixture. We observed no consistent changes in Magnevist or Carboplatin concentrations in the contralateral control side after systemic i.v. and i.c. NG29 administration (Figure 5). In fact, drug concentrations in contralateral tissues felt to negligible levels (≤ 5%) as compared to tumoral tissues from CTL and NG29-treated animals.

### NG29 Increases BTB Permeability and the Uptake of Large Protein Albumin by Rat Gliomas and Peritumoral Tissues

**Figure 6:**
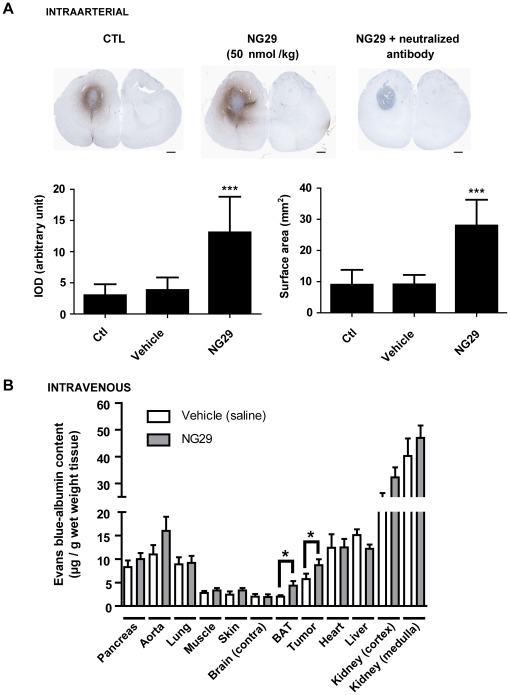
Systemic infusion of NG29 increases permeability and uptake of albumin within peritumoral tissue. (**A**) Direct immunological staining of endogenous albumin in brain tissues from F98-implanted rats treated or not with intracarotid NG29 (50 nmol/kg/min for 5 min). Representative coronal sections of CTL-, vehicle- and NG29-treated rats immunostained with sheep anti-rat albumin HRP conjugated are shown in the upper panels. Scale bar: 1 mm. Histographic representation of integrated optical density (IOD) values (left) and stained surface areas (right) of immunoreactive albumin in respective animal groups (bottom panels). ***p<0.001 vs CTL. (**B**) Semi-quantitative measurement of Evans blue content (mg/g wet weight tissue) in several tissues after systemic intravenous (femoral) injection of saline vehicle or NG29 (5 µmol/kg i.v.) in F98 glioma bearing rats. Data are presented as means ± S.E.M. n = 5 to 7 animals per group. *p<0.05 vs respective vehicle group.

We then looked at whether albumin (∼65 kDa), which is a larger molecule than the CA, could also be delivered to tumor sites by modulating BTB permeability with NG29. Many chemotherapeutic agents (eg, chlorambucil, etoposide, melphalan, vincristine, and paclitaxel) are heavily bound (>90%) to plasma proteins, unabling them to cross the BBB [10]. We thus reasoned that it might be worthwile to use synthetic B1R agonists to facilitate local entry of albumin-bound drugs given that the bound fraction will probably be released into brain tumors in order to maintain equilibrium. We used macroscopic direct albumin immunostaining and EB-staining methods to assess albumin uptake. The results of these experiments are presented in Figure 6 (A and B). Positive albumin immunoreactivity can be seen in brain regions surrounding tumors in control animals, indicating the presence of inflammatory BBB damage (Figure 6A, left upper panel). We observed an increase in extravasated albumin staining in the implanted hemisphere following disruption of the BTB with i.c. NG29 (50 nmol/kg/min for 5 min), with the periphery of tumor having more marked staining than the central portion (Figure 6A, center upper panel). The presence of high interstitial fluid pressure in the core of tumor most likely explains this phenomenom. No staining was observed in control (not shown) and agonist treated sections using the antibody preabsorbed with purified rat albumin antigen (20-fold excess), demonstrating the specificity of the staining patterns observed (Figure 6A, right upper panel). Both the surface area and the intensity of albumin immunostaining (expressed as total IOD) at tumoral sites increased in response to i.c. NG29 (Figure 6A, left and right bottom panels). The co-injection of i.v. NG29 with the albumin-binding dye Evans blue caused brain tumor-specific accumulation of the dye (Figure 6B), suggesting that i.v. administered NG29 homes to brain tumors by initially binding to B1R that are specifically expressed on tumor microvessels. As shown by the IHC staining of albumin, the local induction of plasma albumin extravasation by NG29 was more localized at the invasion site (referred to as BAT) than at the tumor itself (approximatively 2- versus 1.5-fold increase, respectively).

No significant changes in mean arterial blood pressure, heart rate, respiratory rate, or body temperature were observed following the administration of NG29, regardless of administration route and dosing regimen (Figure S3). Hematocrit and arterial pH values also remained in physiological ranges at 1 h post-NG29 administration (data not shown). Thus, the pharmacological modulation of BTB with NG29 agonist appeared to be safe and secure. The apparent lack of toxicity of NG29 was supported by the results of preliminary toxicity analyses consisting of repeated daily bolus injections of single suprapharmacological dose of the peptide NG29 (50 mg/kg, i.v. tail-vein) over a period of three days in normal Fischer rats. Except for a mild elevation of body temperature (control: 37±1 vs treated 39±1C°), which disappeared within few hours (possibly mediated by central release and action of propyretic prostaglandins (Figure 4B)), NG29 had no toxic effects (data not shown).

Taken together, our results show that B1R agonism may be a valuable therapeutic approach for increasing the selective penetration into brain tumors of systemically administered chemotherapeutic drugs or tumor-imaging agents that otherwise have no or limited access to this region. Cognate agonists that are not metabolized in the bloodstream and that temporarily increase BTB permeability may thus be potential adjuvants for optimizing the performance of anticancer drugs used in the treatment of malignant brain cancers.

## Discussion

The salient findings of our study can be summarized as follows: 1) intracranial F98 glioma tumors expressed B1R mRNA and protein; 2) B1R mainly localized in tumor cells (especially in the nuclear compartment) and the tumor vasculature; 3) systemic administration of the metabolically stabilized desArg^9^-bradykinin analogue, NG29, but not the natural agonist LDBK, promoted B1R-mediated transvascular delivery of hydrophilic low- and high-molecular weight soluble macromolecules (Magnevist, Gadomer, Carboplatin and endogenous albumin) through the BTB and their accumulation in tumors; 4) the single permeation of the BTB with NG29 was reversible, was effective by i.v. or i.a. infusion, operated via a COX-mediated mechanism, and appeared relatively safe; and 5) importantly, various human glioblastoma cell lines and glioma patient samples of different grades exhibited high levels of B1R expression compared to their normal cellular/tissue counterparts, identifying the B1R as a potentially relevant biomarker and therapeutic target for brain cancer. Our findings offer preclinical proof of principle that targeting B1R in the brain tumor microenvironment can be a highly effective approach for delivery of chemotherapeutic agents.

Much effort has gone into identifying powerful and selective methods to bypass or transiently breach the BBB/BTB that could be used in chemotherapy protocols [51], [52]. For instance, the chemical modification of drugs and the use of transcellular, receptor-mediated transport mechanisms (known as transcytosis) are possible methods for enhancing drug targeting to brain tumors. However, the clinical benefit of these aproaches has yet to be proved [51], [52]. Other methods to open the BBB/BTB via the paracellular pathway such as the intracarotid infusion of hyperosmolar mannitol or Cereport (Labradimil and formerly called RMP-7), a selective bradykinin B2R agonist, have been shown to be safe, with no associated vasogenic brain edema and apparent neurocognitive function loss, and effective in the adjuvant treatment of experimental and clinical brain tumors [10], [11], [53], [54]. However, both techniques have a number of disadvantages. The osmotic BBB disruption procedure using mannitol is cumbersome and highly invasive, requires general anesthesia, permeabilizes the normal BBB, thus is not fully compatible with certain classes of chemotherapy that are neurotoxic (e.g. taxanes, doxorubicin, cisplatin), and carries a risk of inducing seizures [10]. The BK-B2R-mediated BTB opening using Cereport is transient and lasts only 20 min, even with continuous infusion of the agonist. In addition, it does not work when the agonist is i.v. administered [14]. Cereport also has a narrow therapeutic index and a number of adverse effects, mainly vasodilatation and decreased blood pressure, which limit its practical value [14], [54].

We provide a new mechanistic insight into the design of minimally invasive pharmacological method for inducing local, transient BTB permeability to enhance transvascular transport of drugs intended to detect (namely MRI agents) and to treat brain cancer, thus paving the way for earlier diagnosis and more targeted treatments. The method relies on systemic co-administration of potentially safe, fast acting, synthetic peptide agonists such as NG29 [40] that trigger kinin B1R activity at brain tumor sites. We believe that synthetic biostable peptide B1R agonists, when used as BTB permeability enhancers, may offer a number of potential advantages over other strategies including a) unsurpassed affinity/potency towards human B1R [40], b) expected good safety profile due to their high selectivity and specificity [40], c) a negligible immunogenicity, as with other kinin related peptides of similar size such as Icatibant [55], Cereport [54] and R954 [56] (Sirois P, personal communication), d) a high therapeutic index due to the localized expression of B1R in tumors (Figures 1, 2 and 5) (B1R has virtually no role in normal physiology), e) a lack of tachyphylaxis by receptor desensitization upon agonist exposure [17], which may explain why the BTB-permeability effects of B1R agonists lasted considerably longer than those of the B2R agonists BK and Cereport (Figure 4), f) may not require surgery and general anesthesia if given i.v., and g) do not require chemical modifications to deliver the drug in the CNS.

In addition, our findings showed that there is concordant high-level expression of (vascular and tumoral) B1R on malignant tumors in the preclinical F98 glioma xenograft model (Figure 1), in various human glioblastoma cell lines and in clinical glioma samples (Figure 2), as shown by our RT-PCR, WB, and IHC results. It is to be noted that the high prevalence of B1R protein expression we observed in malignant glioma patient samples may occasionnaly extend to adjacent peritumoral brain regions (Figure 2C), possibly due to the presence of infiltrative tumor cells. Our initial validation of B1R targets in actual high-grade human glioma cells and tumor microvessels underlines the need for further research to assess the potential of B1R agonists in the diagnosis and/or adjuvant chemotherapy of brain tumors. Determining expression levels of expression in human high-grade glioma biopsies may also be valuable for the early prediction of treatment responses to B1R agonists that modulate BTB permeability.

We used high-field MRI to show that the B1R agonist NG29 can induce rapid transvascular delivery and uptake increment (about 1.5-fold higher) of both Magnevist (0.5 kDa) and Gadomer (17 kDa) into intracerebral F98 glioma in syngeneic Fischer rats, as shown by the increase of *T*_1_ MRI intensity signals (Figures 3 and 4). It should be noted that the areas of BTB disruption following intracerebral injection of NG29 corresponded closely with the distribution of B1R in the tumor (Figure 1). Similar efficacy data of NG29 were obtained using a syngeneic rat model of intracerebral metastatic breast cancer (a secondary brain tumor model) with an impeded BTB [57] (Figure S4). The sizes of these MRI agents cover the spectrum of molecular weights of most commonly used antineoplastic agents [9], [52].

Systemic co-administration of NG29 also improved brain tumor uptake profiles (2-fold) of i.v. or i.a. Carboplatin (Figure 5), which is in agreement with the MRI results (Figures 3 and 4). This is in the same range of increase of [^14^C]-Carboplatin levels induced by ic Cereport in RG2 brain tumors [58]. Our results also indicated that NG29 opens the BTB wide enough to allow free and Evans blue bound-albumin (∼65 kDa) to cross the BTB (Figure 5). Good brain tumor penetration of drugs of comparable or smaller dimensions can thus be expected. For instance, B1R agonists could conceivably be used as an add-on treatment to improve glioma immunotherapy aimed at artificially priming the immune system with specific cytokines (ex. IL-12, IFN-β, TNF-α, etc.) that promote antitumor T-cell activity [59], [60], as previously proposed for the selective B2R bradykinin analogue Cereport [61]. Combining B1R agonist with different anticancer drugs may be particularly useful in a multipronged “cocktail” attack of brain tumor cells, which is the gold standard of modern clinical trial designs [62]. However, further work is required to confirm this hypothesis. Overall, our findings demonstrate that the B1R agonist NG29 can be administered i.a. and i.v. in dosages that create preferential extensive extravasation of various drugs in the glioma microenvironment (including central and peripheral portions) while not disturbing the integrity of normal microvessels of organs, such as the normal brain, lung, pancreas, kidney, muscle, skin, heart, liver, and of macrovessels (aortae) (Figure 6). This may be particularly relevant with regards to increasing therapeutic delivery within and most importantly, beyond the edge of the primary tumor in order to gain access to infiltrated tumor foci.

As mentioned, drugs penetrate the BBB/BTB via the paracellular tight junctional and transcellular vesicular pathways, among others [13]. Since albumin and the two CA used in the present study are apparently not transported across the BBB/BTB via any known transcellular pathway, these results would suggest that NG29 mediates the increase in cerebrovascular permeability mostly through a paracellular mechanism. However, in the absence of in vivo results from tests with specific markers of transcellular permeability such as transferrin, insulin, and amino acids [13], it is possible that NG29 increases both paracellular and transcellular permeation across the BTB. Other experiments will be required to clarify this issue.

We have partially deciphered the mechanism underlying the NG29-induced increase in BTB permeability and the delivery of drugs to brain tumors. This may involve direct activation of the B1R at the tumor vasculature causing i) increased blood flow (hyperemia) through arterial/arteriolar vasodilatation, ii) increase in venular tone (venocontriction) leading in both cases to a rise in capillary hydrostatic pressure and/or iii) increased capillary/venule permeability by retraction (fenestration) of the endothelial cells. All of these events may cause in turn, disengagement of tight junctional zones in capillaries/venules and contribute to the opening of the paracellular pathway [29], [63]. In addition, since glioma cells overexpress B1R (Figures 1 and 2) and can secrete capillary permeability factors such as arachidonic acid when activated [64], we cannot exclude the possibility of an indirect mechanism of NG29 passing through the BTB and acting on these cancer cells in the induction of BTB opening, much like the B2R-mediated increase in BTB permeability caused by Cereport [65], [66]. Future studies are necessary to elucidate the exact mechanism(s) responsible for the tumor hyperpermeability responses to NG29. However, our results provide convincing evidence that secondarly released prostanoids play a key role given that pharmacological inhibition of the COX pathway, but not the NOS pathway, repressed NG29 activity. This contrasts with the B2R-mediated increase in BTB permeability in RG2 or F98 glioma-bearing rats, which mainly depends on NO production [34], [67].

As already emphasized for Cereport [68] and mannitol [53], the rate and duration of agent infusion, among other parameters, are to be considered critical factors for successful BTB disruption. Experiments are underway to determine whether prolonged infusion of NG29 could extend much further the opening period of time and retard recovery (closure) of the BBB/BTB. Because of absence of ligand-induced desensitization of B1R [17], a prolongation of signalling pathway initiating endothelial retraction or inhibition of intercellular adhesion may be anticipated. These experiments will most likely be necessary to establish an optimum dosing paradigm and improve therapeutic efficacy. Agonizing the B1R with NG29 to tackle the porosity-permeability problem of the BTB appears relatively safe as there was no abnormality evidence of some forms of hemodynamic and respiratory instabilities. This is in marked contrast with the earlier approach using metabolically protected agonists to activate B2R in order to manipulate the BTB [14], [69]. In fact, under the same experimental conditions, we observed severe undesired effects (non-specific permeability induction, disturbance of respiratory function, hypotension) when the pseudopeptide B2R agonist R523 ([Phe^8^ψ(CH_2_NH)Arg^9^]-BK) [34], [70] was used at the same dose as NG29 (50 nmol/kg/min for 5 min, i.c.) (Figure S4).

One possible concern about the use of exogenous selective B1R (or B2R) kinins as BTB disruptors in adjuvant glioma therapy is that they can potentially act as mitogens with pro-migratory activity (at least in vitro) and may thereby accelerate disease progression [71], [72], [73]. However, some evidence argues against this possibility. First, the proliferation rate of F98 cells cultured in vitro in the presence of LDBK or NG29 (10 µM) over a 96-h exposure period was the same as the control untreated cells, as determined with the MTT and crystal violet colorimetric methods [74] (Figure S5A). Second, and more convincingly, the median and maximum survival of F98 glioma bearing rats following i.a. and i.v. infusion of NG29 at maximal doses tested were the same as those of the vehicle-treated animals (Figure S5B). This is in agreement with the findings of similar survival studies in orthotopic brain tumor xenograft models that used the selective kinin B2R agonist analogue Cereport, which had no growth stimulatory effects on brain tumor cells in vivo (see reviews, [14], [54]).

Lastly, one unanticipated finding of the study was that the B1R are predominantly expressed internally, mainly at the nuclear envelope, in in situ high-grade rat and human glioma tumors (Figures 1 and 2). Elevated B2R levels in the nucleus have recently been documented in the F98 rat glioblastoma model [34]. Elevated nuclear B1R and B2R levels have also been reported in other types of cancer such as malignant pleural mesotheliomas [75] and lung cancer [76]. An emerging concept in the field of GPCRs is that these receptors can function intracellularly on ER/nuclear membranes to promote noncanonical actions in normal physiological as well as disease states [41], [77], [78], [79], [80]. Whether B1R located at the ER/nuclear envelope can mediate intracrine regulation of oncogenic pathways associated with aberrant growth, invasion and survival processes of glioma cells are subjects of investigation in our laboratory.

In conclusion, our results document a novel GPCR signaling mechanism for promoting transient BTB disruption to F98 rat glioma, involving activation of B1R and ensuing production of COX metabolites. Our results also underline the potential value of synthetic B1R agonists as selective BTB modulators for increasing the local delivery of various sized-therapeutic agents to (peri)tumoral sites. Combining chemotherapeutic agents with a B1R agonist may thus be a valuable strategy for improving the effectiveness of the agents against malignant gliomas while possibly minimizing systemic exposure.

## Materials and Methods

### Solid Phase Peptide Synthesis

The peptide kinin agonists LysdesArg^9^BK (H-Lys^0^-Arg^1^-Pro^2^-Pro^3^-Gly^4^-Phe^5^-Ser^6^-Pro^7^-Phe^8^-OH); LDBK), SarLys[dPhe^8^]desArg^9^BK (NG29), and antagonists AcLys[(αMe)Phe^5^,dβNal^7^,Ile^8^]desArg^9^BK (R892) and dArg[Hyp^3^,Thi^5^,dTic^7^,Oic^8^]BK (HOE140), were synthesized on a Pioneer peptide synthesizer using the Fmoc (9-fluorenylmethyoxy-carbonyl) solid-phase chemistry as previously described [40], [70]. Peptide purity (>95%) was assessed by analytical RP-HPLC, and molecular weights were verified by electrospray mass spectrometry using a VG Platform ns 8230E (Waters). Binding affinity (transiently transfected HEK-293T cells) and pharmacological activity (contraction of isolated human umbilical veins) of the peptides on human B1R expressing systems were in agreement with previously reported data from our laboratories, indicating a high level of batch-to-batch consistency [40], [45], [70]. Peptides were stored in powder form at −20°C. Stock solutions (10 mM) of peptides were also prepared in Nanopure water and were stored at −20°C until use. The stock solutions were diluted in sterile 0.9% saline prior to each experiment.

### Animals

Male Fischer 344 rats (250–275 g, Charles River Laboratories, St-Constant, Québec, Canada) were used. Animals were used in full compliance with the Canadian Council of Animal Care guidelines. The protocol was approved by the Committee on the Ethics of Animal Experiments of the University of Sherbrooke (CIPA/CFPA-FMSS).

### Clinical Samples

Fresh human brain tumor tissues were obtained from 41 patients (26 males and 15 females, aged 21–75 years) who underwent therapeutic removal of astrocytic brain tumors between 2004 and 2009 at the Centre Hospitalier Universitaire de Sherbrooke (CHUS). Whenever possible, peritumoral brain samples (comprising infiltrative astrocytomas and normal brain tissues) were also collected. Most specimens were obtained at primary resection. All glioma specimens were classified morphologically and graded by an experienced neuropathologist (AMT), as per WHO criteria. One astrocytoma WHO grade 2 (from a 67 year male) was purchased from BioChain. Post-mortem human brain (frontal or temporal) cortex tissues from white males aged between 36 to 62 years old who had met sudden death resulting from either cardiac arrest (1 case), drowning (1 case), or pulmonary embolism (2 cases)) were obtained from the Maryland Brain collection (Maryland Psychiatric Research Center, Baltimore, MD, USA) and were used as controls. The resected tumors or normal brain tissue specimens were snap-frozen in liquid nitrogen and were stored at −80°C until used for RNA and protein extractions. Other tumors were fixed in 10% buffered formaldehyde for ∼24 h, embedded in paraffin, cut into 3-µm-thick sections, and mounted on silanized slides. For histological confirmation, the sections were stained with haematoxylin and eosin. Written informed consent was obtained from all study participants. The study was carried out with the approval of the research ethic board for human subject of the CHUS.

### Cell Lines and Cultures

The established human glioma cancer cell lines U87-MG (#HTB-14), U138-MG (#HTB-16), U118-MG (#HTB-15), T98G (#CRL1690), LN-229 (#CRL2611), and the F98 rat glioma cells (#CRL-2397) were purchased from American Type Culture Collection (ATCC). These cells were cultured as monolayers in Dulbecco’s modified Eagle medium (DMEM) supplemented with 10% fetal bovine serum (FBS) and 1% penicillin-streptomycin mixture at 37°C in a humidified 5% CO_2_/95% air incubator.

### Glioma Cell Implantation

The procedure of F98 glioma cell implantation was similar to that used in our previous studies [33], [34]. Briefly, F98 glioma cells (1×10^4^ cells in 5 µl) were injected into the region of the right caudate nucleus of the animals under ketamine: xylazine anesthesia (87 mg/kg:13 mg/kg, i.p.) at the following stereotaxic coordinates: 1 mm anterior and 3 mm lateral to bregma, and 6 mm below the external table of the skull. Unless otherwise specified, tumors were allowed to grow for 10 days to mid-stage (approximately 15–20 mm^3^) before the beginning of the experiments. All tumor transplantations were successful as determined by histology and/or MRI.

### RT-PCR

The rats were anesthetized with 2% isoflurane and were transcardially perfused with 60 ml of phosphate-buffered saline (PBS; 0.01 M phosphate-buffered 0.9% NaCl solution, pH 7.6). Brain tumors and control autologous tissues from the contralateral hemisphere were resected from the brains. Resected samples were snap-frozen in liquid nitrogen and were stored at −80°C until use. RNA was extracted and reverse transcribed as previously described [34]. The oligonucleotide primers for amplifying the kinin rat B1R and 18S (internal control), and the length of the expected PCR products (in parentheses) were as follows: B1: F 5′-ACT GTG TCA ACG TCA GGT CAC TGT-3′, R 5′-GAT GCT GAC AAA CAG GTT GGC CTT-3′ (431 bp); 18S : F 5′-GTG CAT GGC CGT TCT TAG TTG GTG-3′, R 5′-CCA TCC AAT CGG TAG TAG CGA CGG-3′ (401 bp). Amplification reaction mixtures contained 1x amplification buffer, 1.5 mM MgCl_2_, 10 mM dNTPs, 400 nM primers, and 1 U of Platinum Pfx DNA polymerase (Invitrogen, Canada). As a negative control, reverse transcriptase was omitted during the initial cDNA synthesis step. The PCR products were quantified using the DNA 1000 kit for the Agilent 2100 Bioanalyzer (Agilent technologies) [34].

### Western Blot

Brain tumors and control autologous subcortical tissue taken from the contralateral hemisphere were resected from the brains under the same conditions as above. Total protein extraction from tissues were prepared by adding RIPA buffer (50 mM Tris–HCl pH 7.4, 150 mM NaCl, 1% NP40, 0.25% deoxycholate, 1 mM EDTA, 1 mM Na_3_VO_4_, 1 mM NaF) containing a protease inhibitor cocktail (Roche, Canada). Brain samples were disrupted using a Potter homogenizer and left on ice for 15 min. Tissue extracts were sonicated twice for 10-sec bursts on ice and then centrifuged at 16,000 g for 15 min at 4°C. Whole-cell lysates were also prepared from semi-confluent human glioma cell line cultures. A lysate of primary normal human astrocytes (NHA) purchased from Sciencell Research Laboratories (Carlsbad, CA) was used as a control. Proteins were quantified using BCA™ (bicinchoninic acid) protein assay kits (Pierce). SDS-PAGE and Western blotting (WB) were performed as previously described [80]. The following primary antibodies and dilutions were used: rabbit anti-B1R antiserum (mix AS434∶0.2 µg/ml; 1∶5,000) (provided by Dr. W Müller-Esterl, University of Mainz, Germany) and mouse monoclonal anti-β-actin antibody (1∶20,000, Sigma-Aldrich). Rabbit anti-mouse (1∶20,000, Sigma-Aldrich) or sheep anti-rabbit (1∶10,000; Serotec) antibody conjugated to horseradish peroxidase was used as secondary antibody. The same techniques were used to extract total proteins from human normal brain and glioma samples for WB. B1R expression was quantified using ImageProPlus 5.1 and was normalized against β-actin expression level. The specificity of the antiserum to B1R (raised against intra- and extra-cellular domains of the human B1R sequence) has been reported elsewhere [70]. On some occasions, the staining pattern seen with the AS434 antiserum was confirmed using two other polyclonal anti-B1R antibodies (recognizing C-terminal domains), namely LS-A799 (1∶1000, LifeSpan) and RC72 (1∶300; a kind gift of Dr. R Couture, Université de Montréal, Canada).

### Immunohistochemistry

F98-implanted rats were deeply anesthetized with ketamine: xylazine on day 10 and were then perfused with PBS, followed by 4% formalin solution. Brains were carefully removed, post-fixed overnight in 4% formalin. A 5-mm-thick coronal brain slice containing the tumor was paraffin embedded, and 3-µm-sections were mounted on positively charged slides for immunochemical localization of B1R. IHC staining was performed with an automated system (Dako Autostainer plus) using the Envision Flex High pH visualization system (Dako). The mix anti-B1R antiserum (AS434) was diluted 1∶800 in blocking buffer and incubated on the brain slides 1 h at room temperature. After washing, secondary sheep anti-rabbit antibody-HRP (1∶100) (Serotec) was added for 1 h at room temperature. Diaminobenzidine (DAB) (Roche) was used as chromogen. Nuclei were counterstained with hematoxylin. An identical IHC staining protocol was used for the human normal brain and glioma biopsies. All the slides were observed under light microscopy (Olympus model BX51). For high-resolution transmission electron microscopy (TEM), tissue samples were processed according to standard techniques [80]. Ultrathin epoxy-embedded sections (∼50 nm) were collected on formvar-coated nickel grids and were immunolabelled with AS434 (1∶20). The grids were then incubated with a goat anti-rabbit gold (10 nm)-conjugated IgG (1∶20) (Sigma-Aldrich) without silver intensification. The sections were then examined using a transmission electron microscope at 120 kV (Hitachi, H-7500). Sections treated with normal (preimmune) rabbit serum were used as negative controls.

### BTB Permeabilization Procedure

The experimental protocol was similar to that used in a previous study [34]. The animals were anesthetized using isoflurane gas (2%) with 1.5 l/min of oxygen. All surgical procedures were performed on a heating pad to avoid per-procedural hypothermia. The caudal vein was catheterized to enable the injection of CA via PE-10 intramedic tubing later connected to a remote-controlled power injector (model PHD 2000, Harvard Apparatus). The right carotid complex was surgically exposed and the external carotid artery was catheterized in a retrograde fashion using PE-50 intramedic tubing such that the tip of the catheter lay just above the bifurcation. This catheter was used to infuse drugs directly into the right hemisphere of the brain via the internal carotid. For the BTB permeabilization procedure, the animals received a single intracarotid infusion of vehicle (sterile 0.9% saline), B1R agonist (LDBK or NG29) (2.5, 10 or 50 nmol/kg/min for 5 min) alone, or NG29 in combination with antagonists for B1R (R892) or B2R (HOE140) (20 nmol/kg/min for 5 min), during MRI scans (see below). Total volume infused was kept constant at 0.5 ml. In other experiments, animals were pretreated with non-selective nitric oxide synthase (NOS) inhibitors (L-NA (5 mg/kg) or L-NAME (20 mg/kg) (Sigma-Aldrich)) or with non-selective cyclooxygenase (COX) inhibitors (Meclofenamate (5 mg/kg) or Indomethacine (2.5 mg/kg) (Sigma-Aldrich)). These inhibitors were given i.v. as a bolus with a 150 µl saline flush via the caudal vein, 30 minutes prior to intracarotid NG29 infusion (10 nmol/kg/min for 5 min). All the inhibitors were dissolved in isotonic saline, except indomethacine, which was dissolved in 75% ethanol prior to dilution. The doses of the inhibitors were selected based on results of previous studies. In some cases, NG29 was also administered via the intrajugular route by a catheter pre-inserted through the right internal jugular vein. Arterial blood pressure was monitored throughout the experimental period with a blood pressure monitor (DigiMed) using the catheter surgically inserted into right femoral artery.

### MRI and Data Post-processing

MRI studies were conducted at the Centre d’Imagerie Moléculaire de Sherbrooke using a Varian 7T small animal scanner (Varian Inc.) equipped with 210/120 mm gradient coils (30 G/cm) and a 63-mm volume RF coil. All MRI measurements were performed according to Côté et al. [34]. For the MRI experiments, the animals were anesthetized with 2% isoflurane (Abott) administered with a face mask. The temperature in the scanner was kept constant at 30°C (with rectal temperature remaining near 37°C) using a warm-air heating system (SA Instruments). Thirty sets of *T*_1_-weighted images were acquired continuously before, during, and after CA injection with a temporal resolution of ∼51 s using the following parameters: TR = 100 ms, TE = 2.49 ms, α = 30°, matrix = 128×128, field of view (FOV) = 35×35 mm^2^, number of averages = 4, and ten contiguous 1.5- mm-thick slices. The MRI experiments were performed in two sessions (without and then with BTB permeabilization procedure) for each animal. This study design allows each animal to act as its own control. The first MRI session was performed with a bolus injection (3 min after the first of 30 scans) of either Magnevist (Gd-DTPA, 0.5 kDa) or Gadomer (∼17 kDa) (143 mM in 500 µl, 1 min duration) (Bayer Schering Pharma AG, Germany) in the lateral tail-vein to determine the extent of BTB permeability in and around the intracranial tumor. The second MRI session was performed using a bolus injection of the same CA immediately following the permeabilization of the BTB with a B1R agonist (see above). The time lags between sessions were 4 h for Magnevist and 12 h for Gadomer. These time lags were selected to ensure complete elimination of the CA from the brain circulation [34].

The image data was processed using MATLAB® (The MathWorks, 2007). Both sets of anatomic MR images were analyzed for the presence or absence of contrast enhancement within and surrounding the tumor areas, and were processed to yield quantitative contrast agent distribution volume (CADV; in mm^3^), which was used as an index of vascular permeability [34]. The relative CADV expressed as a percentage is defined as the normalized ratio between the maximal CADV value determined from the second set of images acquired (CA + BTB permeabilization procedure) and the maximal CADV value determined from the first set of images (CA alone). In separate analyses, the MRI signals were calibrated in terms of the concentration of CA in the brain parenchyma (and jaw muscles) using the precontrast *T*_1_ map and the relaxivity of Magnevist (3.6 mM^−1^s^−1^) and Gadomer (8.74 mM^−1^s^−1^) at 7T.

### ICP-MS of Platinum and Gadolinium

The rats were anesthetized with isoflurane (2%), and either the external carotid artery or the caudal vein was canulated. A mixture of Magnevist (143 mM) and Carboplatin (5 mg/ml/rat; 20 mg/kg) including or not NG29 (i.a.: 250 nmol/kg or i.v.: 5 µmol/kg), was given in a single 1-ml infusion over 15 min. Five minutes after the end of the infusion, anesthetized rats were euthanized by transection of vena cava followed by an intra-cardiac injection of saline (100 ml) in order to flush the blood from the brain. The brain was rapidly removed and placed in physiological saline. The tumors as well as size-related samples of peritumoral tissue and matched tissue located in contralateral hemisphere were resected. These samples were weighed and digested in 2 ml of hydrogen peroxyde (30%)/nitric acid solution for 24 h. Gadolinium (Gd) and platinum (Pt) levels were then measured by inductively coupled plasma-mass spectroscopy (ICP-MS; Elan DRC II, Perkin Elmer).

### Assessment of Vascular Permeability to Albumin

A direct immunohistochemical technique was used for endogenous albumin staining in the evaluation of BBB permeability with i.c. NG29 (see BTB disruption procedure). Slides with 5-µm sections were equilibrated in Tris-buffered saline-0.1% Tween 20 (TBS-T, pH 7.6) for 10 min. They were then incubated with HRP conjugated-sheep anti-rat albumin (Accurate Chemical and Scientific Corporation) diluted 1∶50 in TBS-T overnight at 4°C. After extensive washing, Diaminobenzidine (DAB) (Roche) was used as chromogen. Nuclei were counterstained with hematoxylin. The specificity of the patterns observed was confirmed by preabsorbing the antibody with a 20-fold excess (w/w) of pure rat albumin (Accurate Chemical and Scientific Corporation). Images of whole immunolabelled rat brain tissue sections were acquired using a Nikon Super Coolscan 9000 ED scanner at a resolution of 4000 dpi and analysed with ImagePro 5.1 software. For each animal group (untreated, vehicle control and experimental treated groups; n = 4/group), the integrated optical density (IOD) and surface area (mm^2^) values were calculated from at least four tissue sections per animal. Details of image acquisition, processing and albumin quantification are provided in Methods S1.

Organ-specific NG29-induced extravasation was also assessed in tumor-bearing animals under ketamine: xylazine anesthesia using the semi-quantitative albumin-bound Evans blue method. Immediately after i.v femoral bolus injection of Evans blue (20 mg/kg (in 250 µl), Sigma-Aldrich), the animals were infused with a saline or B1R agonist solution (5 mg/kg (in 500 µl)) over 15 min via a femoral vein catheter. The animals were euthanized after 45 min. Following a systemic perfusion with 250–300 ml of 0.9% saline to remove macromolecules in the circulation, tumor and normal organ specimens (lung, pancreas, kidney, skeletal muscle, skin, aorta, brain, heart ventricle, liver) were excised, minced, and dye extracted by formamide (1 ml per 100 mg tissue) for 72 h. The absorbance of Evans blue at 620 nm was measured with a spectrophotometer (Spectra Max Plus 384, Molecular Devices). The dye concentration in the tissue extracts was calculated from a standard curve of Evans blue.

### Vital Signs and Hemodynamic Measurements

Vital signs (respiratory rate, blood pressure, heart rate, body temperature) and other physiological variables (hematocrit, arterial pH) were monitored during i.a. and i.v. NG29 administration in isoflurane-anesthetized adult F98 glioma-implanted rats. A PE 50 catheter was inserted into the left common carotid artery to continuously monitor the mean arterial pressure and the hearth rate with a Micro-Med transducer (model TDX-300) connected to a blood pressure Micro-Med analyzer (model BPA-100c). Blood samples were withdrawn from a catheter inserted in the left femoral artery to monitor both hematocrit and pH levels.

### Statistical Analysis

Results are expressed as means ± standard error of the mean (S.E.M.) for the specified number (n) of animal tested. Statistical comparisons were analyzed by one-way ANOVA followed by Dunnett’s post hoc test or Student’s t test for unpaired observation when appropriate. *P* values less than 0.05 were considered statistically significant.

## Supporting Information

Figure S1 **IL-1β immunoreactivity in intracranial F98 glioma tissue samples.** IHC analysis was performed 10 days after F98 glioma cells were cerebrally implanted in Fischer rats. 3-µm-thick sections from formalin-fixed, paraffin embedded F98-implanted rat brains were submitted to IHC staining using rabbit anti-rat IL-1β antibody (1∶100; AAR15G AbD Serotec) as described in Materials and Methods. Representative photomicrographs of implanted (left and middle panels) and contralateral (right panel) rat brain hemispheres showing strong immunoreactivity for IL-1β within the main tumor and satellite nodules. Magnification as indicated.
(TIF)

Figure S2 **Specificity of staining with different anti-B1R antibodies assessed by Western blotting.** 50 µg of protein extracts from glioma biopsie specimens (Grade III or IV) were separeted by 9% PAGE and transblotted onto PVDF membranes. Comparative immunodetection of human B1R was performed using rabbit polyclonal anti-B1R antibodies AS434 (from W. Müller-Esterl, Germany), RC72 (from R. Couture, Université de Montréal) or LS-A799 (LifeSpan, BioSciences); final dilutions indicated in parenthesis. All three antibodies detected a major immunoreactive band around 45 kDa. The appearance of other B1R immunoreactive bands on autoradiograms of glioma samples upon longer film exposure suggest that B1R may exist in many post-translational molecular forms. No immunoreactivity was found when membranes were exposed to preimmune serum (right panel). Representative autoradiograms of two independent experiments.
(TIF)

Figure S3 **Collateral consequences of the modulation of BTB permeability with kinin B1R and B2R agonists.** In vivo experiments were conducted under the same experimental conditions as those described for the MRI imaging protocole. The synthetic kinin B2R agonist R523 ([Phe^8^ψ(CH2NH)Arg^9^]-BK) or B1R agonist NG29 (50 nmol/kg/min for 5 min) were i.c. administered in the intracranial F98 glioma-implanted rats. (**A**) Histographic representation of Gadomer uptake (mM) in the contralateral hemispheres and jaw muscles. (**B**) Histographic representation of respiratory rate (breaths/min). *p<0.05, ***p<0.001 versus CTL or vehicle groups. Value represents the mean ± S.E.M obtained with 6 animals. (**C**) Polygraphic (upper) and histographic (bottom) representation of systemic arterial blood pressure (mmHg). ***p<0.001 versus vehicle groups. Value represents the mean ± S.E.M obtained with 9 animals. Note the appearance of severe undesired effects (non-specific permeability induction and disturbance of respiratory function (A; left and right panels), and hypotension (B)) only with use of R523 at equimolar doses of agonists.
(TIF)

Figure S4 **NG29-mediated local BTB disruption in a syngeneic rat model of intracerebral metastatic breast cancer.** Fisher 344 rats were implanted intracranially with MatBIII rat breast cancer cells (1×10^4^ cells/5 ml) as described in Ref. [57]. This rat mammary carcinoma cell line expressed transcripts and proteins of B1R as determined by RT-PCR and WB analyses (data not shown). (**A**) MRI-contrast based detection of the metastatic tumor in a rat brain at day 10 post-inoculation. The presence of the tumor is shown on T1-weighted images of sections 6 and 7 (white arrows). (**B**) Representative axial Magnevist (Gd-DTPA)-enhanced T1-weighted MR images depicting the brain of a MatBIII tumor-bearing rat before and after NG29 treatment (10 nmol/kg/min for 5 min i.c.) (left panels). Note the increase in the signal intensity at the tumor (white arrows). CADV in function of time calculated from the corresponding sets of images (right panel). (**C**) Representative time course of Magnevist (Gd-DTPA) uptake in the ipsilateral (tumor-implanted) and the contralateral hemispheres, before and after treatment with i.c. NG29 (10 nmol/kg/min for 5 min). Histographic representation of average maximal Gd-DTPA concentrations (mM) in the ipsilateral (tumor-implanted) and the contralateral hemispheres following i.c. saline vehicle or NG29 treatment (10 nmol/kg/min for 5 min)). *p<0.05 compared to vehicle-treated ipsilateral groups. Value represents the mean ± S.E.M. obtained with 3 animals.
(TIF)

Figure S5 **NG29 does not modulate proliferation/growth rate of F98 glioma cells both in vitro and in vivo.** (**A**) Cell proliferation assay on F98 cells was determined with colorimetric MTT and Cristal violet assays, as described in Ref. [74]. Cells were seeded in a 96 wells plate at 2,000 cells/well in DMEM media supplemented with 10% FBS for 24 h at 37°C. Cells were then incubated with and without LDBK or NG29 (10 µM) for the indicated times. Data are means ± s.e.m. of 5 to 8 experiments. (**B**) Kaplan-Meier survival curves for F98-glioma-bearing rats after systemic treatment with NG29. NG29 (250 nmol/kg i.c; 5 µmol/kg i.v.) or saline 0.9% was infused over a period of 2 min (250 µl/min) via either the right external carotid or the tail caudal vein. Arrows indicate time of treatment. Note that there are two cycles of treatment for intravenous NG29 on days 7 and 9 after implantation. Survival times, used as an indirect measure of tumor growth, were calculated using the Kaplan-Meier estimation by using the log-rank method in the GraphPad Prism 5.0 software. The median survival times of the vehicle- and NG29-treated groups were similar following intra-arterial (23.5 (n = 8) versus 23.5 days (n = 10)) or intravenous agonist administration (23.5 (n = 8) versus 24.0 days (n = 10)).
(TIF)

Methods S1 **Image acquisition, processing and albumin quantification.**
(DOC)
