# Correction: Induction of Selective Blood-Tumor Barrier Permeability and Macromolecular Transport by a Biostable Kinin B1 Receptor Agonist in a Glioma Rat Model

**DOI:** 10.1371/annotation/6b95427c-645d-4f1b-a648-ceb215129583

**Published:** 2012-06-07

**Authors:** Jérôme Côté, Veronica Bovenzi, Martin Savard, Céléna Dubuc, Audrey Fortier, Witold Neugebauer, Luc Tremblay, Werner Müller-Esterl, Ana-Maria Tsanaclis, Martin Lepage, David Fortin, Fernand Gobeil

The information for references 1 and 2 are incorrect. The correct information is:

1. Department of Pharmacology, Université de Sherbrooke, Sherbrooke, Quebec, Canada

2. Department of Nuclear Medicine and Radiobiology, Université de Sherbrooke, Sherbrooke, Quebec, Canada 

