# Supplementary figures and images for: Induction of Selective Blood-Tumor Barrier Permeability and Macromolecular Transport by a Biostable Kinin B1 Receptor Agonist in a Glioma Rat Model
Source: PLoS One. 2012 May 21;7(5):e37485. doi: 10.1371/journal.pone.0037485 (PMC3357387; doi:10.1371/journal.pone.0037485)

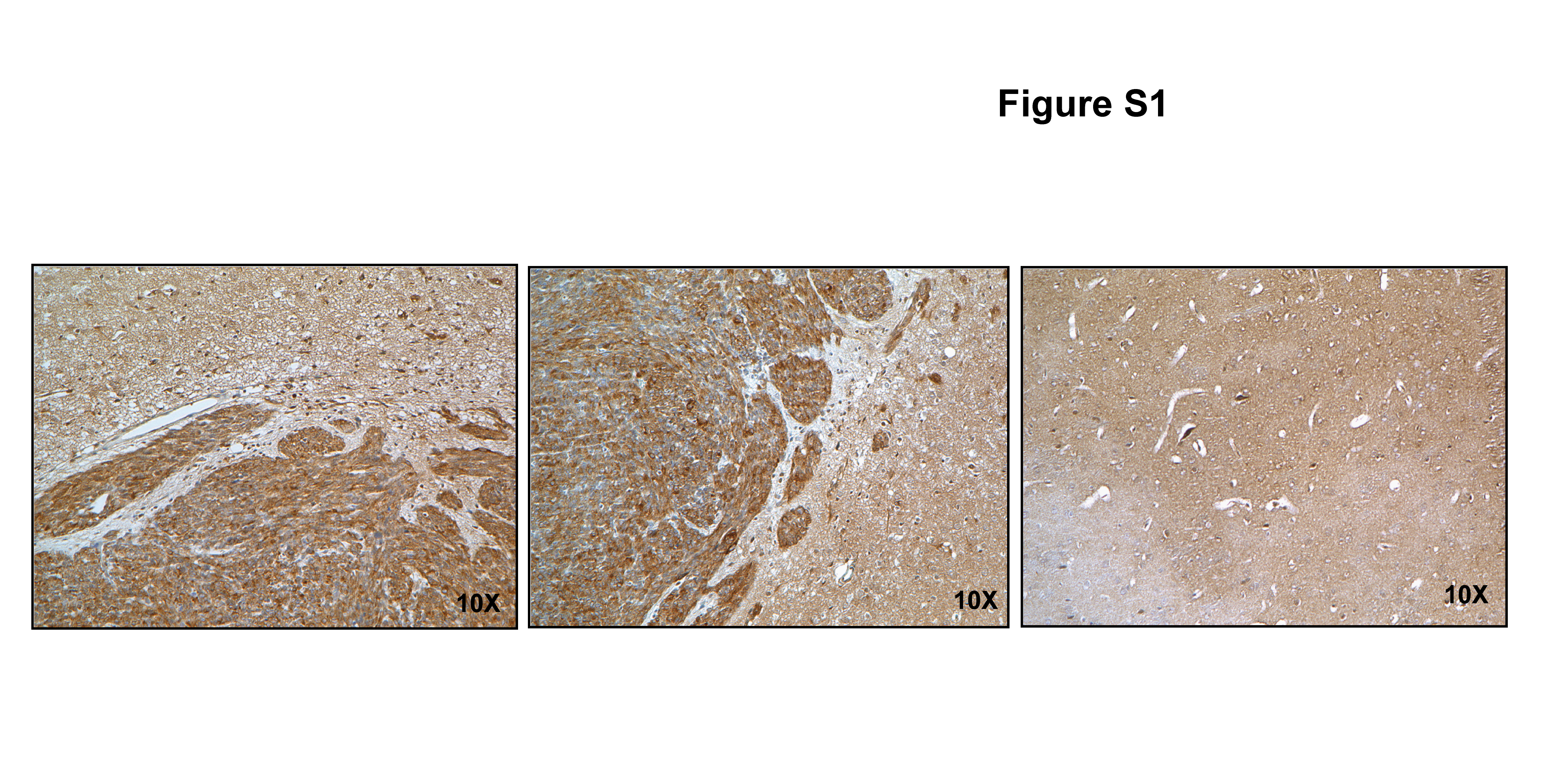

Supplement: Figure S1 — IL-1β immunoreactivity in intracranial F98 glioma tissue samples. IHC analysis was performed 10 days after F98 glioma cells were cerebrally implanted in Fischer rats. 3-µm-thick sections from formalin-fixed, paraffin embedded F98-implanted rat brains were submitted to IHC staining using rabbit anti-rat IL-1β antibody (1∶100; AAR15G AbD Serotec) as described in Materials and Methods. Representative photomicrographs of implanted (left and middle panels) and contralateral (right panel) rat brain hemispheres showing strong immunoreactivity for IL-1β within the main tumor and satellite nodules. Magnification as indicated. (TIF) [file pone.0037485.s001.tif]

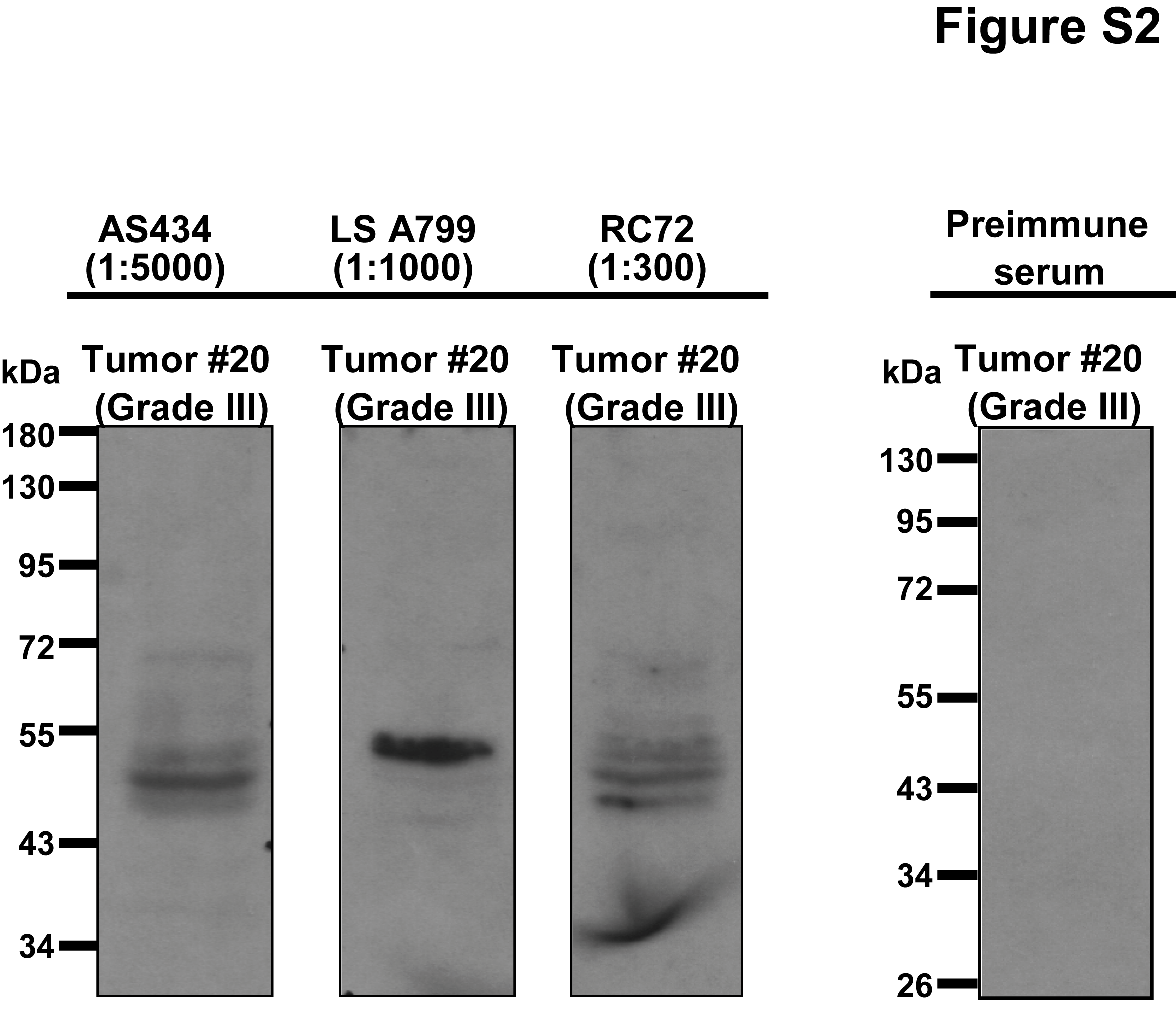

Supplement: Figure S2 — Specificity of staining with different anti-B1R antibodies assessed by Western blotting. 50 µg of protein extracts from glioma biopsie specimens (Grade III or IV) were separeted by 9% PAGE and transblotted onto PVDF membranes. Comparative immunodetection of human B1R was performed using rabbit polyclonal anti-B1R antibodies AS434 (from W. Müller-Esterl, Germany), RC72 (from R. Couture, Université de Montréal) or LS-A799 (LifeSpan, BioSciences); final dilutions indicated in parenthesis. All three antibodies detected a major immunoreactive band around 45 kDa. The appearance of other B1R immunoreactive bands on autoradiograms of glioma samples upon longer film exposure suggest that B1R may exist in many post-translational molecular forms. No immunoreactivity was found when membranes were exposed to preimmune serum (right panel). Representative autoradiograms of two independent experiments. (TIF) [file pone.0037485.s002.tif]

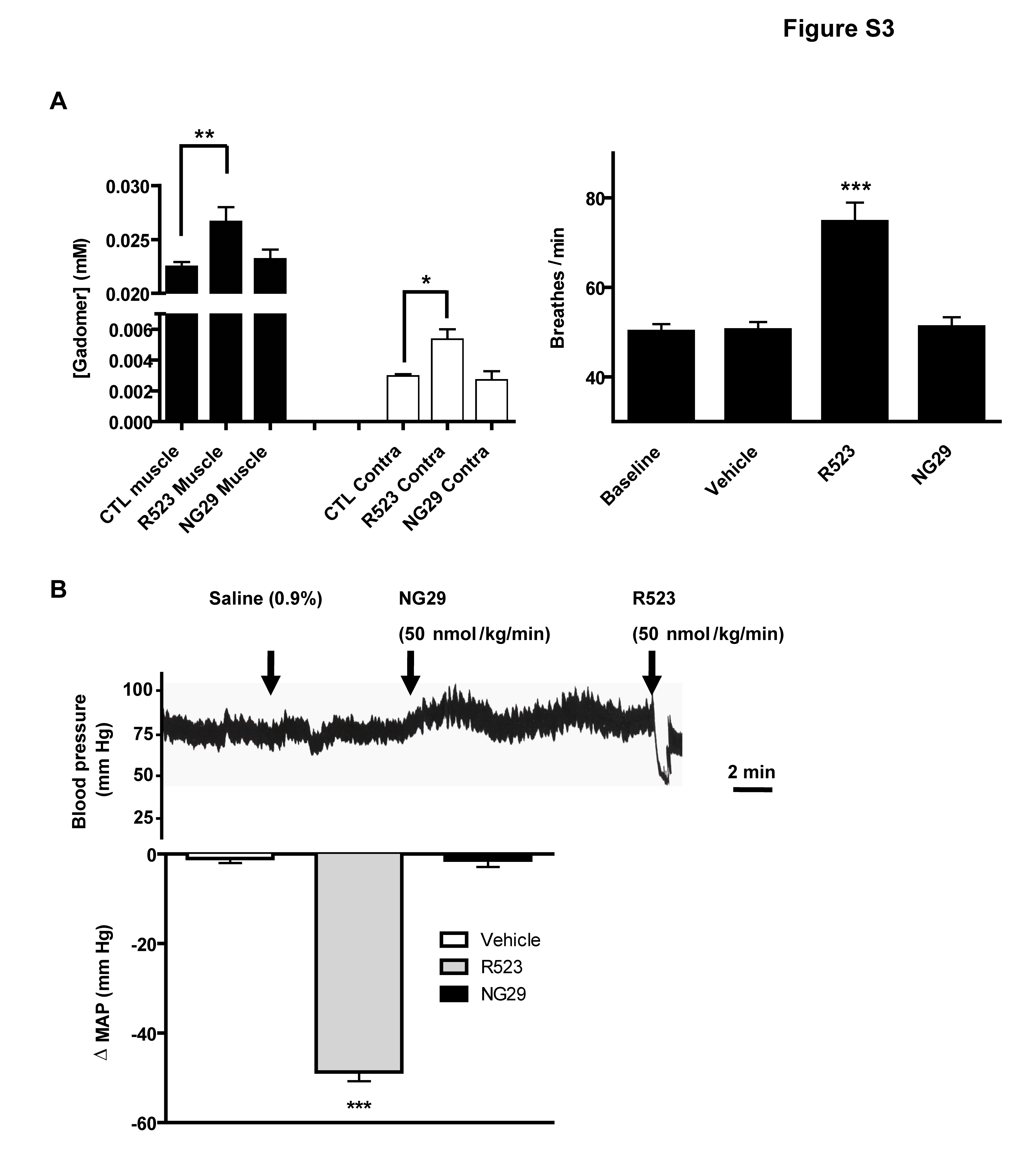

Supplement: Figure S3 — Collateral consequences of the modulation of BTB permeability with kinin B1R and B2R agonists. In vivo experiments were conducted under the same experimental conditions as those described for the MRI imaging protocole. The synthetic kinin B2R agonist R523 ([Phe8ψ(CH2NH)Arg9]-BK) or B1R agonist NG29 (50 nmol/kg/min for 5 min) were i.c. administered in the intracranial F98 glioma-implanted rats. (A) Histographic representation of Gadomer uptake (mM) in the contralateral hemispheres and jaw muscles. (B) Histographic representation of respiratory rate (breaths/min). *p<0.05, ***p<0.001 versus CTL or vehicle groups. Value represents the mean ± S.E.M obtained with 6 animals. (C) Polygraphic (upper) and histographic (bottom) representation of systemic arterial blood pressure (mmHg). ***p<0.001 versus vehicle groups. Value represents the mean ± S.E.M obtained with 9 animals. Note the appearance of severe undesired effects (non-specific permeability induction and disturbance of respiratory function (A; left and right panels), and hypotension (B)) only with use of R523 at equimolar doses of agonists. (TIF) [file pone.0037485.s003.tif]

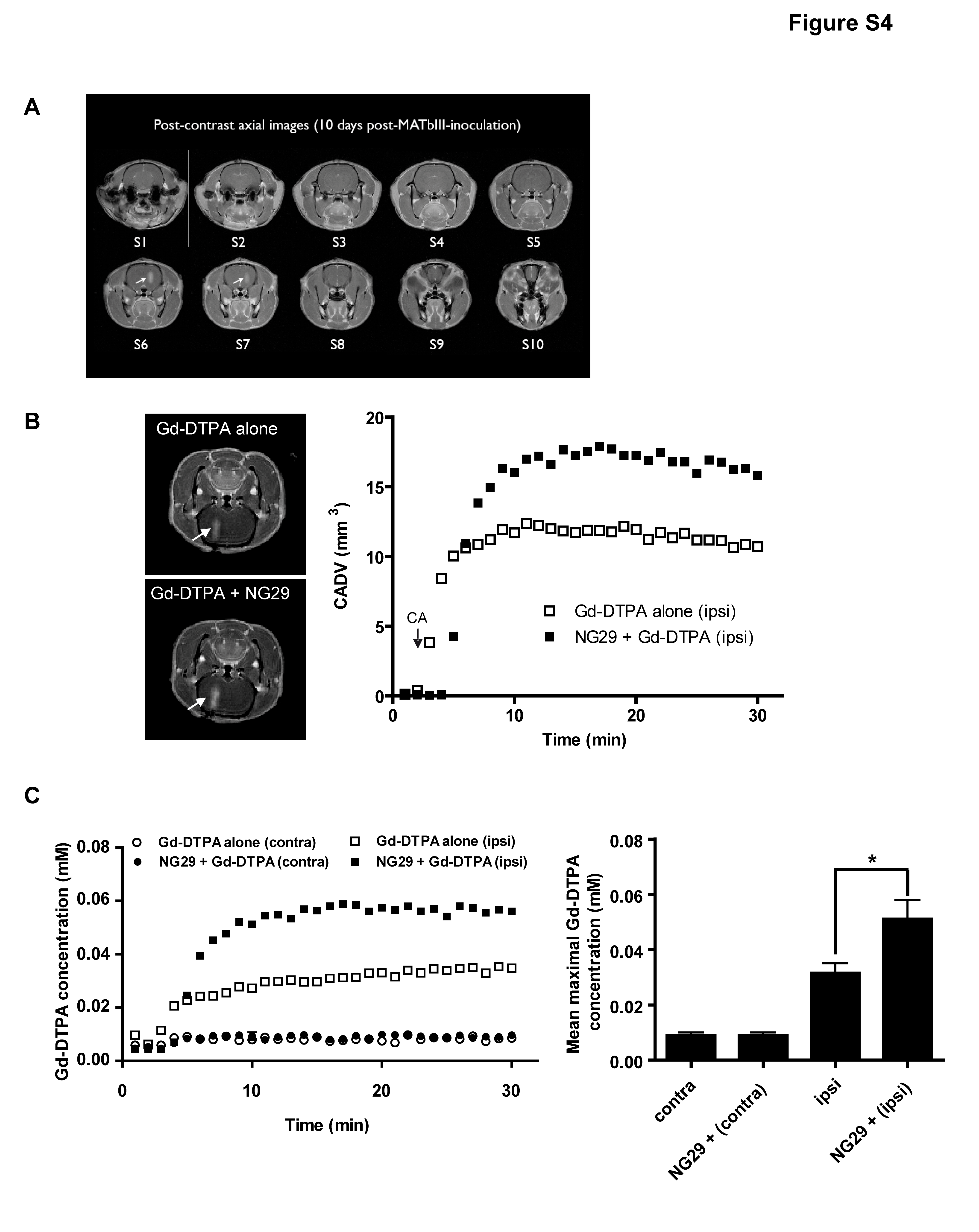

Supplement: Figure S4 — NG29-mediated local BTB disruption in a syngeneic rat model of intracerebral metastatic breast cancer. Fisher 344 rats were implanted intracranially with MatBIII rat breast cancer cells (1×10^4^ cells/5 ml) as described in Ref. [57]. This rat mammary carcinoma cell line expressed transcripts and proteins of B1R as determined by RT-PCR and WB analyses (data not shown). (A) MRI-contrast based detection of the metastatic tumor in a rat brain at day 10 post-inoculation. The presence of the tumor is shown on T1-weighted images of sections 6 and 7 (white arrows). (B) Representative axial Magnevist (Gd-DTPA)-enhanced T1-weighted MR images depicting the brain of a MatBIII tumor-bearing rat before and after NG29 treatment (10 nmol/kg/min for 5 min i.c.) (left panels). Note the increase in the signal intensity at the tumor (white arrows). CADV in function of time calculated from the corresponding sets of images (right panel). (C) Representative time course of Magnevist (Gd-DTPA) uptake in the ipsilateral (tumor-implanted) and the contralateral hemispheres, before and after treatment with i.c. NG29 (10 nmol/kg/min for 5 min). Histographic representation of average maximal Gd-DTPA concentrations (mM) in the ipsilateral (tumor-implanted) and the contralateral hemispheres following i.c. saline vehicle or NG29 treatment (10 nmol/kg/min for 5 min)). *p<0.05 compared to vehicle-treated ipsilateral groups. Value represents the mean ± S.E.M. obtained with 3 animals. (TIF) [file pone.0037485.s004.tif]

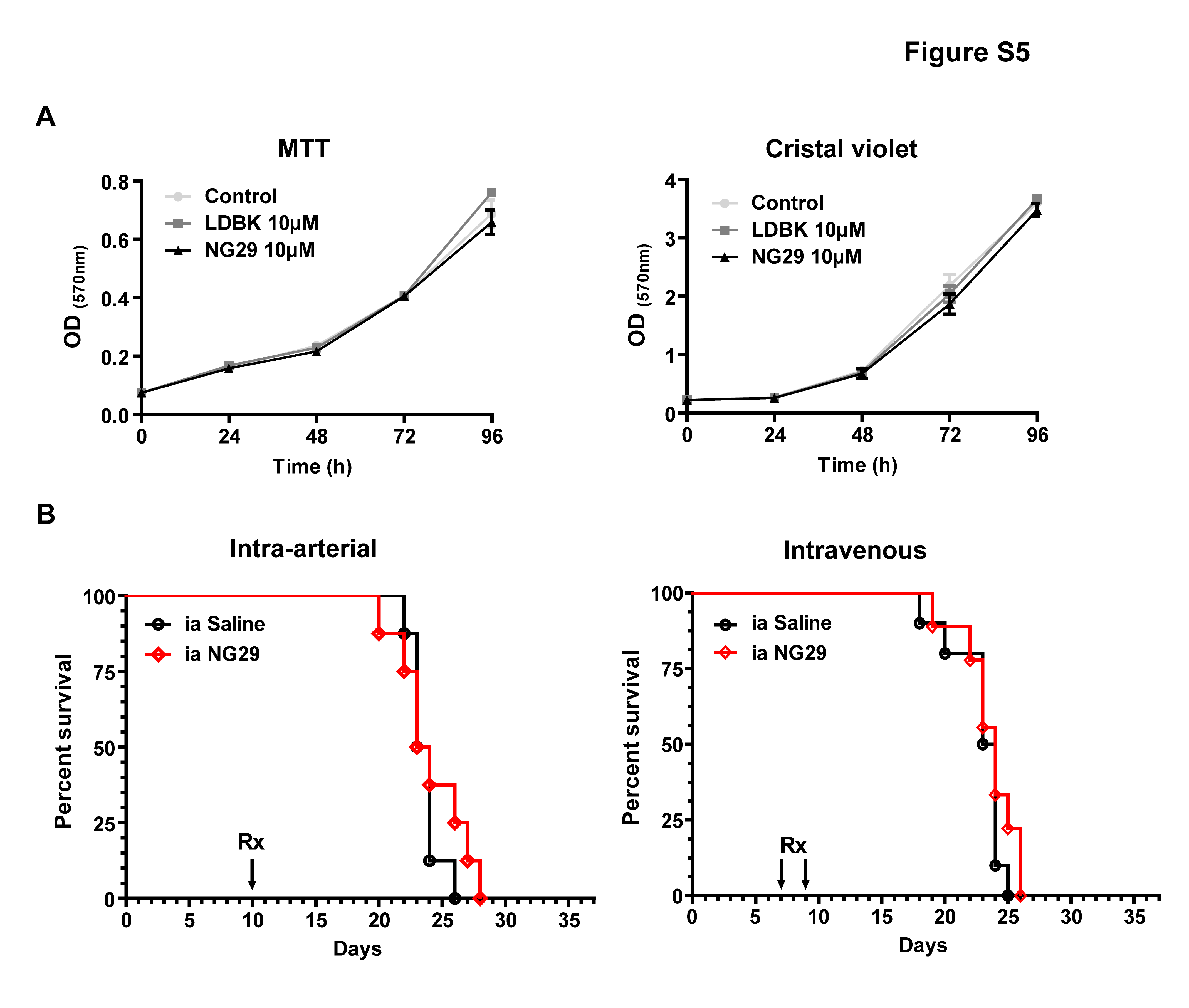

Supplement: Figure S5 — NG29 does not modulate proliferation/growth rate of F98 glioma cells both in vitro and in vivo. (A) Cell proliferation assay on F98 cells was determined with colorimetric MTT and Cristal violet assays, as described in Ref. [74]. Cells were seeded in a 96 wells plate at 2,000 cells/well in DMEM media supplemented with 10% FBS for 24 h at 37°C. Cells were then incubated with and without LDBK or NG29 (10 µM) for the indicated times. Data are means ± s.e.m. of 5 to 8 experiments. (B) Kaplan-Meier survival curves for F98-glioma-bearing rats after systemic treatment with NG29. NG29 (250 nmol/kg i.c; 5 µmol/kg i.v.) or saline 0.9% was infused over a period of 2 min (250 µl/min) via either the right external carotid or the tail caudal vein. Arrows indicate time of treatment. Note that there are two cycles of treatment for intravenous NG29 on days 7 and 9 after implantation. Survival times, used as an indirect measure of tumor growth, were calculated using the Kaplan-Meier estimation by using the log-rank method in the GraphPad Prism 5.0 software. The median survival times of the vehicle- and NG29-treated groups were similar following intra-arterial (23.5 (n = 8) versus 23.5 days (n = 10)) or intravenous agonist administration (23.5 (n = 8) versus 24.0 days (n = 10)). (TIF) [file pone.0037485.s005.tif]
