# Supplementary material for: Induction of Selective Blood-Tumor Barrier Permeability and Macromolecular Transport by a Biostable Kinin B1 Receptor Agonist in a Glioma Rat Model
Source: PLoS One. 2012 May 21;7(5):e37485. doi: 10.1371/journal.pone.0037485 (PMC3357387; doi:10.1371/journal.pone.0037485)
Supplement: Methods S1 — Image acquisition, processing and albumin quantification. (DOC) [file pone.0037485.s006.doc]

**Methods S1**

**Image acquisition, processing and albumin quantification.** Images of whole immunolabelled rat brain tissue sections were acquired using a Nikon Super Coolscan 9000 ED scanner at a resolution of 4000 dpi and analysed with ImagePro 5.1 software. The grid matrix of each of the images was calibrated so as to give a yield of 6 µm/pixel. To separate the immunostain (brown stain) from the haematoxylin stain (blue stain) the cube-based method of the color segmentation operation was applied. The brownish stain was selected with a sensitivity of 4 (maximum 5). Surface areas of the selected immunostained regions were calculated using the count/mesure tool and region outlines were saved. The images were next transformed to a 8 bit gray scale TIFF format to which the saved outlines were applied. After spatial and intensity of light calibration of the images, the immunohistochemically stained area and the integrated optical density (IOD) of the labelled reaction, defined by the antigen-antibody complex was obtained. For each animal group, IOD and Surface area values were calculated from at least 4 tissue sections per animal and exported to a spreadsheet in order to perform the statistical analysis.
